# Microwave Assisted Synthesis, Characterization and Biological Activities of Ferrocenyl Chalcones and Their QSAR Analysis

**DOI:** 10.3389/fchem.2019.00814

**Published:** 2019-11-26

**Authors:** Dinesh K. Yadav, Parshant Kaushik, Pankaj, Virendra S. Rana, Deeba Kamil, Dilip Khatri, Najam A. Shakil

**Affiliations:** ^1^Division of Agricultural Chemicals, ICAR-Indian Agricultural Research Institute, New Delhi, India; ^2^Division of Nematology, ICAR-Indian Agricultural Research Institute, New Delhi, India; ^3^Division of Plant Pathology, ICAR-Indian Agricultural Research Institute, New Delhi, India; ^4^PI Industries, Udaipur, India

**Keywords:** ferrocenyl chalcones, antifungal activity, *Sclerotium rolfsii*, *Alternaria solani*, root-knot nematode, *Meloidogyne incognita*, QSAR

## Abstract

A new microwave method (MM) has been developed for the synthesis of a series of 16 substituted ferrocenyl chalcones using acetylferrocene (**1**) with different aldehydes (**2a-2p**) and comparing it with conventional method (CM). The synthesized compounds were characterized by various spectroscopic techniques *viz* IR, HR-MS, ^1^H NMR, and ^13^C NMR. The time required for completion of reaction in MM varied from 1 to 5 min as compared to CM which required 10–40 h. All the synthesized compounds were screened for antifungal activity against *Sclerotium rolfsii* and *Alternaria solani*. *In vitro* fungicidal activity revealed that compound **3o** (ED_50_ = 23.24 mg L^−1^) was found to be most active against *S. rolfsii*. But in case of *A. solani*, compound **3c** (ED_50_ = 29.9 mg L^−1^) showed highest activity. The nematicidal activity revealed that the compound **3b** was more potent with LC_50_ values of 10.67, 7.30, and 4.55 ppm at 24, 48, and 72 h, respectively. 2D-Quantitative Structural Activity Relationship (2D-QSAR) analysis of these ferrocenyl chalcones was carried out by developing three different models namely Partial Least Squares (PLS, Model 1), Multiple Linear Regression (MLR, Model 2) and Principal Component Regression (PCR, Model 3). Statistical significance and predictive ability of these models were assessed by internal and external validation and also verified by leave one-out cross-validation. QSAR study revealed that MLR for *S. rolfsii* (*r*^2^ = 0.999, *q*^2^ = 0.996), PLS for *A. solani* (*r*^2^ = 0.934, *q*^2^ = 0.749) and PCR for *M. incognita* (*r*^2^ = 0.878, *q*^2^ = 0.772) were the best model. The physico-chemical parameters were calculated using VLife MDS 4.6 software. QSAR study could be employed for structure optimization to achieve better activity.

## Introduction

Chalcones and their corresponding heterocyclic analogs were reported to exhibit several biological activities such as antibacterial, antifungal, insecticidal, nematicidal, anti-oxidant, antiplasmodial, antitumor, and anthelmintic due to occurrence of highly reactive unsaturated carbonyl moiety in skeleton (Vibhute and Baseer, 2003; Alam, 2004; Bag et al., 2009; Kalirajan et al., 2009; Shakil et al., 2010, 2011, 2013; Caboni et al., 2016). Although chalcones do exhibit promising activity against various pests, but have not been explored in agricultural practices to that extent due to their poor water solubility, which can be overcome by making suitable formulations. However, to develop formulations, there is a need to incorporate auxiliaries, adjuvants, other materials and also the process of developing formulation is cumbersome as well as costly to end users.

Ferrocene based organometallic compounds are important because of their interference to the biological system. Due to greater stability, easy availability of derivatives, and favorable electrochemical properties, ferrocene derivatives have been used prominently (Nolte and Salmain, 2008). Wu et al. (2002) synthesized ferrochloroquine, which is the ferrocenyl analog of chloroquine and reported its antimalarial activity against *Plasmodium falciparum*.


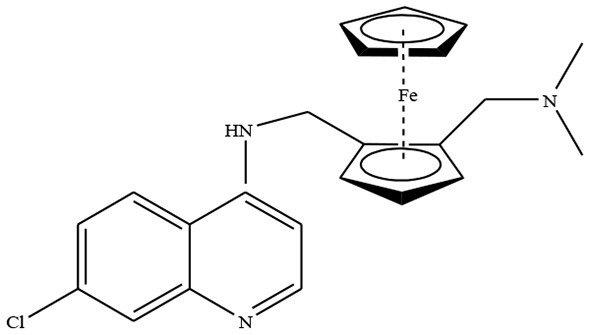


**Ferrochloroquine**

In recent time, microwave heating has taken an incontestable place in analytical and organic laboratories practice as a very effective and non-polluting method of activation (Arends et al., 1997). Synthesis through microwave, has several advantages *viz*., shorter reaction times, higher yields, ease of manipulation, and lower costs. Rate enhancement of organic reactions is due to the superheating of the solvent (Gedye et al., 1986; Mayo et al., 2002).

The early blight disease caused by *Alternaria solani* is most threatening and severely influenced the tomato productivity across the world and accounts for 35–78% loss in fruit yield (Grigolli et al., 2011; Song et al., 2011). Another widely distributed, soil-borne fungal pathogen, affecting a large number of horticultural, and agricultural crops is *Sclerotium rolfsii*. In tomato, they cause tomato blight resulting in damping-off and collar rot (Abeysinghe, 2009).

Plant parasitic nematodes are most important soil borne pests that cause damage to almost all species of crop plant. *Meloidogyne* spp. (Root-knot nematodes) are the most destructive pathogens in terms of yield loss due to their broad host range i.e., vegetables, cereals, pulses, etc. (Abad et al., 2008). *M. incognita* is one of the important pest of vegetables belonging to Solanaceous crop (brinjal, tomato, chili etc.). They have been reported to cause extensive damage to the tune of 20–32% (Jain et al., 2007).

Chalcones and their derivatives are conventionally known to possess biological activity, but their poor water solubility hamper their utility, both in pharmaceuticals and agricultural field. Through this study, efforts have been made to improve the solubility/bioavailability of substituted chalcones by the introduction of ferrocene group. Also, new microwave method was developed for synthesizing these molecules in reduced time as compared to conventional method. Synthesized ferrocenyl chalcones have been evaluated for their fungicidal and nematicidal activity against *S. rolfsii, A. solani*, and *M. incognita*. QSAR analysis was carried out to recognize the molecular properties which effect the fungicidal and nematicidal activities the most.

## Experimental

### Chemicals and Instruments

Acetylferrocene and different benzaldehydes were procured from Aldrich. All other solvents and chemicals were of analytical grade and were used as received unless otherwise noted. Reactions were observed by thin layer chromatography (TLC) on pre-coated Merck silica gel 60F_254_, 200 mm thick aluminum sheets and the spots were visualized under UV-light. Reverse-phase UFLC SHIMADZU, C-18 Shim-pack column (5 μm, 4.6 × 250 mm) with PDA detector using isocratic solvent system (methanol-water: 98:2) was used for determining the purity of synthesized ferrocenyl chalcones. ^1^H-NMR and ^13^C-NMR studies were carried out on JEOL 400 MHz Spectrospin spectrometer instrument, data were processed using Delta software and tetramethylsilane (TMS) was used as internal standard.

High Resolution Mass Spectrometry (HRMS) was performed by AB SCIEX Triple TOF™ 5,600^+^ equipped with TurboIonSpray (TIS), SCIEX ExionLC, and PDA detector. C-18 column (2.7 μm, 4.6 × 100 mm) was used for separation of compounds. The column was eluted with methanol and water (98:2, v/v) with 0.1% formic acid at a flow rate of 0.3 mL/min. The column oven temperature was set at 40°C. Infrared (IR) spectra were recorded on Bruker alpha FT-IR spectrophotometer, values were expressed as υ_max_ cm^−1^. Melting point apparatus was used for determination of melting points. ED_50_ and LC_50_ values were calculated by SPSS statistical package. All computational activities were performed by using Vlife MDS QSAR plus 4.6 software in ASUS VivoBook (windows 10 OS and Intel Core i5).

### Synthesis

#### Conventional Method (CM)

The synthesis of the ferrocenyl chalcones series was done by the base-catalyzed Claisen-Schmidt reaction according to the method reported in literature. The general method of synthesis is reproduced below (Wu et al., 2002).

##### General method for the synthesis of ferrocenyl chalcones

**Scheme 1:**
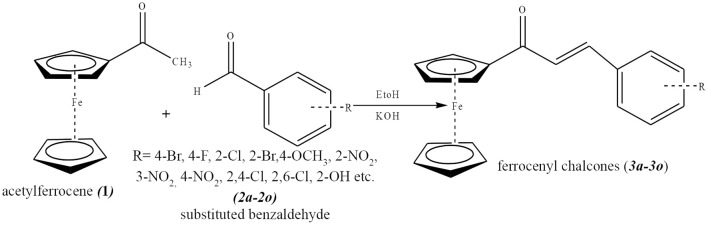
General method for the synthesis of ferrocenyl (Wu et al., 2002).

A series of ferrocenyl chalcones [except 3-hydroxybenzaldehyde (**2i**), 4-hydroxybenzaldehyde (**2p**)] was synthesized as follows: Acetylferrocene (684 mg, 3 mmol) and KOH (0.2 g) were dissolved in ethanol (5 mL) in a round bottom flask and stirred at room temperature for 10 min, followed by drop wise addition of ethanolic solution of equimolar amounts of different benzaldehydes (**2a**-**2o**), (3 mmol, 5 mL) with continuous stirring. The stirring ranged between 1 and 40 h for different reactions at room temperature (Scheme 1). The reaction was monitored by TLC in ethyl acetate: hexane, (1:4 solvent systems). After completion of reaction, the reaction mixture was neutralized with 2 M HCl to get the dark red/red/orange/chocolate precipitate. The precipitate was separated by filtration and washed with cold water. In case of absence of precipitate, the solution was extracted with ethyl acetate (30 mL × 3). The organic layer was dried with anhydrous Na_2_SO_4_ and solvent removed using rotary evaporator to give a viscous residue. The crude product was purified by column chromatography on silica gel using hexane-ethyl acetate as eluent of increasing polarity. The desired compound was eluted in 10% ethyl acetate: hexane. Characterization of all compounds was done by IR, HR-MS, ^1^H-NMR, and ^13^C-NMR (Supplementary Data Sheets 1–3).

##### Method for the synthesis of 3i and 3p (ferrocenyl chalcones)

**Scheme 2:**
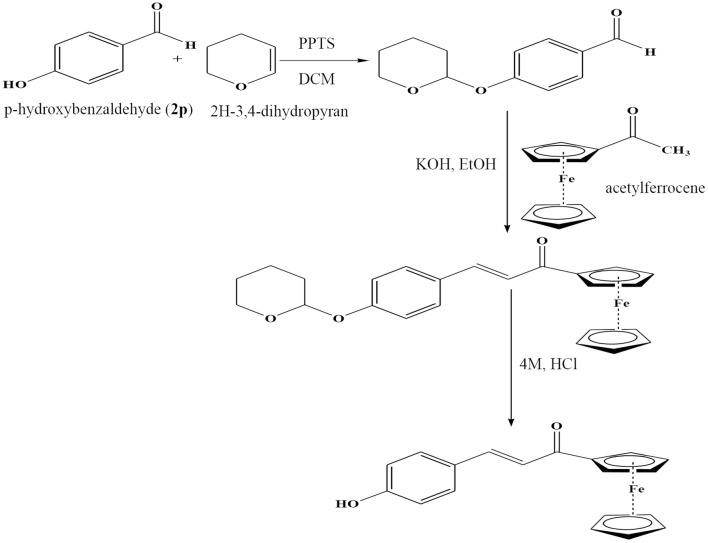
Method for the synthesis of **3p**, ferrocenyl (Wu et al., 2002).

3i and ***3p*** were synthesized as follows: The hydroxy derivatives of benzaldehydes (**2i** and **2p**) (366 mg, 3 mmol), 2H-3, 4-dihydropyran (8 mmol), and pyridinium p-toluenesulfonate (0.2 mmol) were taken in DCM (10 mL) and kept for stirring at room temperature for 4 h (Scheme 2). After completion of reaction, crude tetrahydropyranyl ether was obtained as yellow solid by washing the reaction mixture with Na_2_CO_3_ (1 M, 20 mL × 2), followed by removal of solvent under vacuum. These protected benzaldehydes (**2i and 2p**) were then treated with acetylferrocene as per the method described above to get the corresponding ferrocenyl chalcones.

##### Microwave method (MM)

Equimolar amounts of acetylferrocene (100 mg, 0.438 mmol) in 5% ethanolic KOH (5 mL) with minimum ethanolic solution of the different benzaldehydes were added in microwave vial (10 mL). The vial was placed in microwave synthesis reactor (Anton Paar, Monowave 300) with the conditions; Temp. 100°C, rpm-600, and time 1–5 min. Monitoring of reaction was carried out by TLC in ethyl acetate: hexane (1:4 solvent systems). Reaction mixture was worked up as described above.

### Spectral Analysis of Synthesized Ferrocenyl Chalcones

#### (2E)-1-Ferrocenyl-3-phenyl-prop-2-en-1-one (3a)

It was obtained as a red colored solid in 81% yield; m.p.:140–142°C, R_f_: 0.50 (ethyl acetate: hexane, 1:4). IR (cm^−1^): 1,645 (CO), 1,593 (C = C). ^1^H-NMR (400 MHz, ACN-d_3_): δ 4.22 (5H, s, H-6″), 4.63 (2H, s, H-3″ & H-4″), 4.94 (2H, s, H-2″ & H-5″), 7.29 (1H, d, J = 16, H-α), 7.45 (3H, m, H-3′, H-4′ & H-5′), 7.67 (1H, d, J = 16, H-β), 7.76 (2H, d, J = 8, H-2′ & H-6′).^13^C- NMR (400 MHz, DMSO-d_6_): δ 69.65 (C-2″ & C-5″), 69.78 (C-6″), 72.74 (C-3″ & C-4″), 80.66 (C-1″), 123.64 (C-α), 128.61 (C-3′ & C-5′), 128.89 (C-2′ & C-6′), 130.11 (C-4′), 134.90 (C-1′), 139.75 (C-β), 191.96 (CO). HR-MS for C_19_H_16_FeO [M+H]^+^ *m/z:* Calcd 317.0623; Observed 317.0639.

#### (2E)-1-Ferrocenyl- 3-(4-bromophenyl)-prop-2-en-1-one (3b)

It was obtained as an orange colored solid in 87% yield; m.p.:160–162°C, R_f_: 0.42 (ethyl acetate: hexane, 1:4). IR (cm^−1^): 1,649 (CO), 1,591 (C = C). ^1^H-NMR (400 MHz, ACN-d_3_): δ 4.22 (5H, s, H-6″), 4.64 (2H, s, H-3″ & H-4″), 4.93 (2H, s, H-2″ & H-5″), 7.28 (1H, d, J = 16, H-α), 7.61 (2H, d, J = 8, H-2′ & H-6′), 7.63 (1H, d, J = 12, H-β), 7.68 (2H, d, J = 8, H-3′ & H-5′). ^13^C-NMR (400 MHz, DMSO-d_6_): δ 69.70 (C-2″ & C-5″), 69.80 (C-6″), 72.83 (C-3″ & C-4″), 80.59 (C-1″), 123.45 (C-α), 124.43(C-4′), 130.55 (C-2′ & C-6′), 131.82 (C-3′ & C-5′), 134.24 (C-1′), 138.41 (C-β), 191.88 (CO). HR-MS for C_19_H_15_BrFeO [M+H]^+^ *m/z:* Calcd 394.9728; Observed 394.9739.

#### (2E)-1-Ferrocenyl-3-(4-fluorophenyl)-prop-2-en-1-one (3c)

It was obtained as a red colored solid in 79% yield; m.p.:138–140°C, R_f_: 0.43 (ethyl acetate: hexane, 1:4). IR (cm^−1^): 1,649 (CO), 1,589 (C = C). ^1^H-NMR (400 MHz, ACN-d_3_): δ 4.22 (5H, s, H-6″), 4.63 (2H, s, H-3″ & H-4″), 4.93 (2H, s, H-2″ & H-5″), 7.20 (2H, d, J = 8, H-3′ & H-5′), 7.22 (1H, d, J = 16, H-α), 7.65 (1H, d, J = 16, H-β), 7.81 (2H, d, J = 8, H-2′ & H-6′).^13^C-NMR (400 MHz, DMSO-d_6_): δ 69.28 (C-2″ & C-5″), 69.99 (C-6″), 72.95 (C-3″ & C-4″), 80.87 (C-1″), 115.62 (C-3′ & C-5′), 123.79 (C-α), 130.60 (C-2′ & C-6′), 131.07 (C-1′), 138.34 (C-β), 163.64 (C-4′),192.45 (CO). HR-MS for C_19_H_15_FFeO [M+H]^+^ *m/z:* Calcd 335.0529; Observed 317.0544.

#### (2E)-1-Ferrocenyl-3-(2-chlorophenyl)-prop-2-en-1-one (3d)

It was obtained as a chocolate colored solid in 80% yield; m.p.:125–126°C, R_f_: 0.60 (ethyl acetate: hexane, 1:4). IR (cm^−1^): 1,653 (CO), 1,591 (C = C). ^1^H-NMR (400 MHz, ACN-d_3_): δ 4.19 (5H, s, H-6″), 4.52 (2H, s, H-3″ & H-4″), 4.62 (2H, s, H-2″ & H-5″), 6.64 (1H, d, J = 16, H-α), 7.24 (1H, d, J = 16, H-β), 7.42, 7.47, 7.51, 7.53 (4H, m, H-3′, H-4′, H-5′, & H-6′).^13^C-NMR (400 MHz, DMSO-d_6_): δ 68.24 (C-2″ & C-5″), 69.12 (C-6″), 72.99 (C-3″ & C-4″), 80.35 (C-1″), 124.22 (C-α), 125.89 (C-5′), 127.20 (C-6′), 128.56 (C-3′), 130.6 (C-4′), 133.73 (C-2′), 136.61 (C-1′), 139.85 (C-β), 192.87 (CO). HR-MS for C_19_H_15_ClFeO [M+H]^+^ *m/z:* Calcd 351.0233; Observed 351.0231.

#### (2E)-1-Ferrocenyl- 3-(2-bromophenyl)-prop-2-en-1-one (3e)

It was obtained as a red colored solid in 83% yield; m.p.:120–122°C, R_f_: 0.50 (ethyl acetate: hexane, 1:4). IR (cm^−1^): 1,653 (CO), 1,595 (C = C). ^1^H-NMR (400 MHz, ACN-d_3_): δ 4.24 (5H, s, H-6″), 4.66 (2H, s, H-3″ & H-4″), 4.94 (2H, s, H-2″ & H-5″), 7.24 (1H, d, J = 16, H-α), 7.31 (1H, t, H-4′), 7.44 (1H, t, H-5′), 7.69 (1H, d, J = 8, H-6′), 7.96 (1H, d, J = 4, H-3′), 7.97 (1H, d, J = 16, H-β).^13^C-NMR (400 MHz, DMSO-d_6_): δ 69.71 (C-2″ & C-5″), 69.86 (C-6″), 72.99 (C-3″ & C-4″), 80.35 (C-1″), 125.09 (C-2′), 126.51 (C-α), 128.20 (C-5′), 128.56 (C-4′), 131.66 (C-6′), 133.23 (C-3′), 134.21 (C-1′)137.14 (C-β), 191.70 (CO). HR-MS for C_19_H_15_BrFeO [M+H]^+^ *m/z:* Calcd 394.9728; Observed 394.9724.

#### (2E)-1-Ferrocenyl-3-(4-methoxyphenyl) prop-2-en-1-one (3f)

It was obtained as an orange colored solid in 82% yield; m.p.:150–152°C, R_f_: 0.28 (ethyl acetate: hexane, 1:4). IR (cm^−1^): 1,645 (CO), 1,585 (C = C). ^1^H-NMR (400 MHz, ACN-d_3_): δ 3.84 (3H, s, 4′, Ar-OCH_3_), 4.21 (5H, s, H-6″), 4.60 (2H, s, H-3″ & H-4″), 4.92 (2H, s, H-2″ & H-5″), 6.99 (2H, d, J = 8, H-3′ & H-5′), 7.17 (1H, d, J = 16, H-α), 7.63(1H, d, J = 16, H-β), 7.72 (2H, d, J = 8, H-2′ & H-6′).^13^C-NMR (400 MHz, DMSO-d_6_): δ 55.34 (4′, Ar-OCH_3_), 69.54 (C-2″ & C-5″), 69.70 (C-6″), 72.48 (C-3″ & C-4″), 80.87 (C-1″), 114.35 (C-3′ & C-5′), 121.26 (C-α), 127.56 (C-1′), 130.37 (C-2′ & C-6′), 139.65 (C-β), 160.92 (C-4′), 191.90 (CO). HR-MS for C_20_H_18_FeO_2_ [M+H]^+^ *m/z:* Calcd 347.0729; Observed 347.0749.

#### (2E)-1-Ferrocenyl-3-(4-N, N-dimethylaminophenyl) prop-2-en-1-one (3g)

It was obtained as a chocolate colored solid in 78% yield; m.p.:140–142°C, R_f_: 0.40 (ethyl acetate: hexane, 1:4). IR (cm^−1^): 1,639 (CO), 1,562 (C = C). ^1^H-NMR (400 MHz, ACN-d_3_): δ 3.01 (6H, s, 4′, Ar-N(CH_3_)_2_), 4.19 (5H, s, H-6″), 4.57 (2H, s, H-3″ & H-4″), 4.90 (2H, s, H-2″ & H-5″), 6.76 (2H, d, J = 8, H-3′ & H-5′), 7.08 (1H, d, J = 16, H-α), 7.58 (2H, d, J = 8, H-2′ & H-6′), 7.61 (1H, d, J = 16, H-β).^13^C-NMR (400 MHz, DMSO-d_6_): δ 40.65 (4′, Ar-N(CH_3_)_2_), 69. 68 (C-2″ & C-5″), 69.97 (C-6″), 72.28 (C-3″ & C-4″), 81.05 (C-1″), 111.79 (C-3′ & C-5′), 117.80 (C-α), 122.62 (C-1′), 130.17 (C-2′ & C-6′), 141.24 (C-β), 151.72 (C-4′), 192.92 (CO). HR-MS for C_21_H_21_FeNO [M+H]^+^ *m/z:* Calcd 360.1045; Observed 360.1049.

#### (2E)-1-Ferrocenyl-3-(3,4,5-trimethoxyphenyl) prop-2-en-1-one (3h)

It was obtained as a chocolate colored solid in 80% yield; m.p.:145–146°C, R_f_: 0.30 (ethyl acetate: hexane, 1:4). IR (cm^−1^): 1,643 (CO), 1,579 (C = C). ^1^H-NMR (400 MHz, ACN-d_3_): δ 3.77 (3H, s, 4′, Ar-OCH_3_), 3.90 (6H, s, 3′&5′, Ar-OCH_3_), 4.22 (5H, s, H-6″), 4.63 (2H, s, H-3″ & H-4″), 4.97 (2H, s, H-2″ & H-5″), 7.05 (2H, s, H-2′& H-6′), 7.21 (1H, d, J = 16, H-α), 7.60 (1H, d, J = 16, H-β). ^13^C-NMR (400 MHz, DMSO-d_6_): δ 55.31 (3′&5′, Ar-OCH_3_), 60.10 (4′, Ar-OCH_3_), 69.60(C-2″ & C-5″), 69.76(C-6″), 72.59 (C-3″ & C-4″), 80.95 (C-1″), 106.17 (C-2′ & C-6′), 122.82 (C-α), 129.56 (C-1′), 130.37 (C-4′), 140.21 (C-β), 153.07 (C-3′ & C-5′), 191.79 (CO). HR-MS for C_22_H_22_FeO_4_ [M+H]^+^ *m/z:*Calcd 407.0940; Observed 407.0931.

#### (2E)-1-Ferrocenyl- 3-(3-hydroxyphenyl)-prop-2-en-1-one (3i)

It was obtained as a dark red colored solid in 81% yield; m.p.:120–122°C, R_f_: 0.37 (ethyl acetate: hexane, 1:4). IR (cm^−1^): 1,647 (CO), 1,585 (C = C). ^1^H-NMR (400 MHz, ACN-d_3_): δ 4.22 (5H, s, H-6″), 4.63 (2H, s, H-3″ & H-4″), 4.94 (2H, s, H-2″ & H-5″), 5.53 (1H, s, 3′, Ar-OH), 7.29 (1H, d, J = 16, H-α), 7.38 (4H, m, H-2′ H-4′, H-5′ & H-6′), 7.63 (1H, d, J = 16, H-β).^13^C-NMR (400 MHz, DMSO-d_6_): δ 69.51 (C-2″ & C-5″), 69.77 (C-6″), 72.74 (C-3″ & C-4″), 80.28 (C-1″), 116.21 (C-2′), 118.05 (C-4′), 122.21 (C-6′), 123.91 (C-α), 129.86 (C-5′), 136.15(C-1′), 139.62 (C-β), 157.17 (C-3′), 192.80 (CO). HR-MS for C_19_H_16_FeO_2_ [M+H]^+^ *m/z:* Calcd 333.0572; Observed 333.0588.

#### (2E)-1-Ferrocenyl-3-(4-benzyloxyphenyl) prop-2-en-1-one (3j)

It was obtained as an orange colored solid in 82% yield; m.p.:140–142°C, R_f_: 0.34 (ethyl acetate: hexane, 1:4). IR (cm^−1^): 1,645 (CO), 1,579 (C = C). ^1^H-NMR (400 MHz, ACN-d_3_): δ 4.21 (5H, s, H-6″), 4.60 (2H, s, H-3″ & H-4″), 4.92 (2H, s, H-2″ & H-5″), 5.16 (2H, s, 4′, Ar-OCH_2−_C_6_H_5_), 7.06 (2H, d, J = 20, H-3′ & H-5′), 7.16 (1H, d, J = 16, H-α), 7.41 (5H, s, Ar-OCH_2−_C_6_H_5_), 7.46 (2H, d, H-2′ & H-6′), 7.66 (1H, d, J = 16, H-β).^13^C-NMR (400 MHz, DMSO-d_6_): δ 69.53 (C-2″ & C-5″), 69.69 (C-6″), 72.48 (C-3″ & C-4″), 77.45 (4′, Ar-OCH_2_-Ar), 80.85 (C-1″), 115.19 (C-3′ & C-5′), 121.37 (C-α), 127.73 (C-1′, C-2′ & C-6′), 127.90 (4′, Ar-OCH_2_-Ar, C-2″ ′ & C-6″ ′), 128.44 (4′, Ar-OCH_2_-Ar, C-4^″′^), 130.36 (4′, Ar-OCH_2_-Ar, C-3^″′^ & C-5^″′^), 136.59 (4′, Ar-OCH_2_-Ar, C-1^″′^), 139.57 (C-β), 159.89 (C-4′), 191.94 (CO). HR-MS for C_26_H_22_FeO_2_ [M+H]^+^ *m/z:* Calcd 423.1042; Observed 423.1045.

#### (2E)-1-Ferrocenyl-3-(2-nitrophenyl) prop-2-en-1-one (3k)

It was obtained as dark viscous liquid in 71% yield; R_f_: 0.25 (ethyl acetate: hexane, 1:4). IR (cm^−1^): 1,648 (CO), 1,594 (C = C). ^1^H-NMR (400 MHz, ACN-d_3_): δ 4.24 (5H, s, H-6″), 4.55 (2H, s, H-3″ & H-4″), 4.94 (2H, s, H-2″ & H-5″), 7.24 (1H, d, J = 16, H-α), 8.01 (1H, d, J = 16, H-β), 7.77 (3H, m, H-4′, H-5′ H-4′ & H-6′), 8.04 (1H, d, J = 12, H-3′). ^13^C-NMR (400 MHz, DMSO-d_6_): δ 69.64 (C-2″ & C-5″), 69.95 (C-6″), 72.91 (C-3″ & C-4″), 80.88 (C-1″), 125.68 (C-α), 126.81 (C-3′), 128.62 (C-6′), 129.98 (C-5′), 131.79 (C-1′), 134.12 (C-4′), 137.21 (C-β), 148.98 (C-2′), 192.25 (CO). HR-MS for C_19_H_15_FeNO_3_ [M+H]^+^ *m/z:* Calcd 362.0474; Observed 362.0476.

#### (2E)-1-ferrocenyl-3-(3-nitrophenyl) prop-2-en-1-one (3l)

It was obtained as a red colored solid in 80% yield; m.p.:175–176°C, R_f_: 0.31 (ethyl acetate: hexane, 1:4). IR (cm^−1^): 1,651 (CO), 1,595 (C = C). ^1^H-NMR (400 MHz, ACN-d_3_): δ 4.23 (5H, s, H-6″), 4.66 (2H, s, H-3″ & H-4″), 4.97 (2H, s, H-2″ & H-5″), 7.43 (1H, d, J = 16, H-α), 7.68–7.74 (2H, m, H-5′ & H-6′), 8.13 (1H, d, H-4′). 8.24 (1H, d, J = 16, H-β), 8.60 (1H, s, H-2′). ^13^C-NMR (400 MHz, DMSO-d_6_): δ 69.53 (C-2″ & C-5″), 69.82 (C-6″), 72.97 (C-3″ & C-4″), 80.41 (C-1″), 122.81 (C-2′), 124.21 (C-4′), 126.28 (C-α), 130.28 (C-5′), 134.79 (C-6′), 136.88 (C-1′), 137.38 (C-β), 148.40 (C-3′), 191.81 (CO). HR-MS for C_19_H_15_FeNO_3_ [M+H]^+^ *m/z:* Calcd 362.0474; Observed 362.0480.

#### (2E)-1-Ferrocenyl-3-(4-nitrophenyl) prop-2-en-1-one (3m)

It was obtained as a violet colored solid in 82% yield; m.p.:180–182°C, R_f_: 0.33 (ethyl acetate: hexane, 1:4). IR (cm^−1^): 1,653 (CO), 1,597 (C = C). ^1^H-NMR (400 MHz, ACN-d_3_): δ 4.24 (5H, s, H-6″), 4.68 (2H, s, H-3″ & H-4″), 4.96 (2H, s, H-2″ & H-5″), 7.39 (1H, d, J = 16, H-α), 7.72 (1H, d, J = 16, H-β), 7.97 (2H, d, J = 8, H-2′ & H-6′), 8.27 (2H, d, J = 8, H-3′ & H-5′). ^13^C-NMR (400 MHz, DMSO-d_6_): δ 69.48 (C-2″ & C-5″), 69.85 (C-6″), 72.98 (C-3″ & C-4″), 80.67 (C-1″), 123.77 (C-3′ & C-5′), 125.72 (C-α), 129.20 (C-2′ & C-6′), 136.91 (C-β), 141.21 (C-1′), 148.67 (C-4′),192.41 (CO). HR-MS for C_19_H_15_FeNO_3_ [M+H]^+^ *m/z:* Calcd 362.0474; Observed 362.0478.

#### (2E)-1-Ferrocenyl-3-(2,4-dichlorophenyl)-prop-2-en-1-one (3n)

It was obtained as dark viscous liquid in 76% yield; R_f_: 0.60 (ethyl acetate: hexane, 1:4). IR (cm^−1^): 1,651 (CO), 1,584 (C = C). ^1^H-NMR (400 MHz, ACN-d_3_): δ 4.24 (5H, s, H-6″), 4.66 (2H, s, H-3″ & H-4″), 4.88 (2H, s, H-2″ & H-5″), 7.29 (1H, d, J = 16, H-α), 7.31 (1H, d, J = 8, H-5′), 7.33 (1H, d, J = 8, H-6′), 7.49 (1H, s, H-3′), 7.66 (1H, d, J = 16, H-β).^13^C-NMR (400 MHz, DMSO-d_6_): δ 69.46 (C-2″ & C-5″), 69.85 (C-6″), 73.23 (C-3″ & C-4″), 79.83 (C-1″), 123.28 (C-α), 128.75 (C-5′), 130.62 (C-6′), 132.05 (C-3′), 132.34 (C-2′), 132.86 (C-1′), 133.78 (C-4′), 139.36 (C-β), 191.17 (CO). HR-MS for C_19_H_14_Cl_2_FeO [M+H]^+^ *m/z:* Calcd 384.9843; Observed 384.9813.

#### (2E)-1-Ferrocenyl-3-(2,6-dichlorophenyl)-prop-2-en-1-one (3o)

It was obtained as dark viscous liquid in 83% yield; R_f_: 0.60 (ethyl acetate: hexane, 1:4). IR (cm^−1^): 1,654 (CO), 1,586 (C = C). ^1^H-NMR (400 MHz, ACN-d_3_): δ 4.23 (5H, s), 4.66 (2H, s, H-3″ & H-4″), 4.93 (2H, s, H-2″ & H-5″), 7.45 (1H, d, J = 16, H-α), 7.21–7.35 (1H, m, H-4′), 7.66 (1H, d, J = 16, H-β),7.94–7.98 (2H, m, H-3′& H-5′).^13^C-NMR (400 MHz, DMSO-d_6_): δ 69.72 (C-2″ & C-5″), 70.00 (C-6″), 73.14 (C-3″ & C-4″), 80.35 (C-1″), 124.24 (C-α), 128.07 (C-3′& C-5′), 129.03 (C-4′), 132.51 (C-2′ & C-6′), 135.22 (C-1′), 139.48 (C-β), 192.35 (CO). HR-MS for C_19_H_14_Cl_2_FeO [M+H]^+^ *m/z:* Calcd 384.9843; Observed 384.9856.

#### (2E)-1-Ferrocenyl- 3-(4-hydroxyphenyl)-prop-2-en-1-one (3p)

It was obtained as an orange colored solid in 80% yield; m.p.:115–120°C, R_f_: 0.45 (ethyl acetate: hexane, 1:4). IR (cm^−1^): 1,645 (CO), 1,571 (C = C). ^1^H-NMR (400 MHz, ACN-d_3_): δ 4.21 (5H, s, H-6″), 4.60 (2H, s, H-3″ & H-4″), 4.92 (2H, s, H-2″ & H-5″), 5.52 (1H, s, 4′, Ar-OH), 7.08 (2H, d, J = 8, H-3′ & H-5′), 7.17 (1H, d, J = 16, H-α), 7.63 (1H, d, J = 16, H-β), 7.70 (2H, d, J = 8, H-2′ & H-6′).^13^C-NMR (400 MHz, DMSO-d_6_): δ 69.59 (C-2″ & C-5″), 69.89 (C-6″), 72.28 (C-3″ & C-4″), 80.37 (C-1″), 116.35 (C-3′ & C-5′), 120.78 (C-α), 127.06 (C-1′), 127.37 (C-2′ & C-6′), 139.80 (C-β), 156.92 (C-4′), 192.37 (CO). HR-MS for C_19_H_16_FeO_2_ [M+H]^+^ *m/z:* Calcd 333.0572; Observed 333.0579.

### Biological Assay

#### Antifungal Activity

Two fungi, *A. solani ITCC* 4632 and *S. rolfsii* ITCC 6474 were procured from Indian Type Culture Collection (ITCC) center, Division of Plant Pathology, ICAR-Indian Agricultural Research Institute, New Delhi-110012, India and kept at 27°C for at least 4–7 days on Potato Dextrose Agar (PDA) slant. Sub-culturing was done in petriplates and before doing the antifungal activity.

*In vitro* antifungal activity of the all synthesized compounds was carried out on PDA medium by using poisoned food technique (Nene and Thapliyal, 1979). A stock solution of 10,000 ppm of each compound was prepared in DMSO (Dimethylsuphoxide). Five test concentrations *viz*. 100, 50, 25, 12.5, and 6.25 mg L^−1^ were prepared from stock solution by serial dilution. The commercial fungicides, Hexaconazole 5% SC (*S. rolfsii*) and Mancozeb 75% WP (*A. solani)* were taken as positive control. Percentage inhibition was calculated by Abott's formula (Abbott, 1925).

Corrected % inhibition (IC) was calculated by given formula

$$\begin{array}{l} \text{IC}=\left(\text{I}-\text{CF}\right)/\left(100-\text{CF}\right)\times \;100, \end{array}$$

Where, I = Percentage inhibition, CF = (90-C)/C × 100, 90 is the diameter (mm) of the petriplate and C is the growth of the fungus (mm) in control.

ED_50_ (mg L^−1^) values (Effective Dose for 50% inhibition) were calculated using SPSS statistical Package (v16.0).

### Nematicidal Activity

*In vitro* nematicidal activity of the all synthesized compounds was carried by water screening method. The root galls were obtained from *M. incognita* infected plants collected from the glass house of Division of Nematology, ICAR-IARI, New Delhi and were incubated for 2–3 days at 25–30°C for hatching of eggs. After hatching, the number of J2s per 1 mL of aliquot were calculated and diluted to 100 J2s per mL. Synthesized ferrocenyl chalcones (12 mg) were weighed separately and dissolved in 0.5 mL of DMSO and 24.5 mL distilled water with Tween 80 (3.0%) to get a stock solution of 500 ppm. Five test concentrations *viz*. 125, 62.5, 31.25 15.62, and 7.81 mg L^−1^ were prepared from stock solution by serial dilution. 1 mL of aliquot (100 J2s/mL) was taken in 24 well culture plate to which 1 mL of test concentrations was added (Figure 3). All treatments were carried out in triplicates separately for 24, 48, and 72 h. Mortality was recorded after 24, 48, and 72 h using a stereoscopic binocular microscope. The percent mortality and the corrected percent mortality was calculated using Abbott's formula.

Corrected mortality % = T – C/100 – C × 100 (where, T = Total mortality in treatment; C = Total mortality in control).

LC50 value (ppm) was calculated for each compound with the help SPSS statistical Package (v16.0) using ASUS VivoBook laptop.

### Quantitative Structure Activity Relationship (QSAR)

QSAR study was performed by using negative logarithm of experimental LC_50_ (ppm) [pLC_50_ = –log (LC_50_)] as dependent variable and 2D descriptors (Table 5) as independent variables. ChemDraw Ultra 7.0 software was used for drawing 2D structures of compounds which were converted to mol structures. To describe several features of molecular structure 239 2D descriptors were taken and composed of structural, thermodynamic, electronic and spatial descriptors, e.g., element count, atomic valence connectivity index (chiV), refractivity, estate number, chain path count, retention index (chi), path cluster, semi-empirical path count, molecular cluster, topological index, molecular weight, and logP. The various descriptors were calculated by energy minimization and geometry optimization through batch energy minimization method using Merck Molecular Force Field (MMFF) by taking RMS gradient (0.01), number of cycles (max. 1,000) and distance dependent dielectric (1). Various Baumann alignment-independent (AI) descriptors were also calculated. All computational activities were performed by using Vlife MDS QSAR plus 4.6 software in ASUS VivoBook (windows 10 OS and Intel Core i5).

#### Training and Test Set

Entire data of 16 compounds was divided into two sets i.e., training set (11 compounds) and test set (5 compounds) with the help of sphere exclusion method (Hudson et al., 1996). Unicolumn statistics was done to establish the accuracy of selection of training and test sets (Tables 2B, 3B, 4B) as maximum value of the training set was higher than that of the test set and the minimum value of the training set was lower than that of the test set.

## Results and Discussion

### Chemistry

Ferrocenyl chalcones were prepared by condensation of acetylferrocene with different benzaldehydes both by conventional and microwave methods. It was found that the yield was higher in microwave method (78–92%) as compared to conventional method (71–87%) (Table 1). In MM, reaction was completed in 1–5 min as compared to CM requiring 10–40 h (Table 1). Peaks at δ 6.64–7.45 (1H, d, J = 16 Hz, H-α) and δ 7.24–8.24 (1H, d, J = 16 Hz, H-β) as double doublets for two protons with J = 16 Hz each, were characteristic peaks of olefinic bond in ^1^H-NMR spectrum of all the compounds (**3a**-**3p**) and established the condensation of carbonyl moiety of acetylferrocene with carbonyl moiety of different benzaldehydes. In ^13^C-NMR, the peaks at δ 117.80–126.28 (C-α), 136.91–141.24 (C-β) for HC = CH and at δ 191.17–192.92 for CO were prominent for all the compounds. In all compounds, the higher chemical shifts values of H-α and C-α as compared to H-β and C-β was due to carbonyl moiety which polarizes the C = C double bond (Solcaniova et al., 1980). Stretching of (CO) at 1,639–1,654 and (C = C) at 1,562–1,595 cm^−1^ in IR spectra justified the NMR data.

**Table 1:** Comparison of reaction time and yield (%) of Microwave (MM) and Conventional methods.

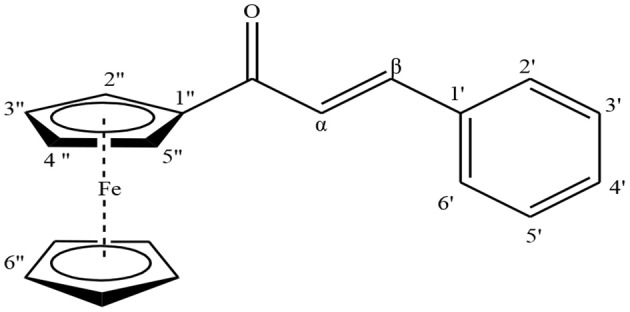					
		**Conventional method**		**Microwave method**	
**Compound**	***R***	**Rxn. Time and RT (h)**	**Yield (%)**	**Rxn. Time, Temp**. **−100°C and 600 RPM (Min)**	**Yield (%)**
3a	H	26	81	1	88
3b	4-Br	10	87	1	92
3c	4-F	27	79	2	89
3d	2-Cl	12	80	1	85
3e	2-Br	24	83	3	90
3f	4-OCH_3_	15	82	1	90
3g	4-N(CH_3_)	40	78	5	84
3h	3,4,5- OCH_3_	30	80	3	85
3i	3-OH	24	81	3	86
3j	4-benzyloxy	21	82	3	91
3k	2-NO_2_	40	71	4	78
3l	3-NO_2_	16	80	2	88
3m	4-NO_2_	20	82	2	87
3n	2,4- Cl	36	76	3	88
3o	2,6- Cl	36	83	3	91
3p	4-OH	24	80	4	88

### Bio-efficacy Evaluation

#### Antifungal Activity

**Figure 1:**
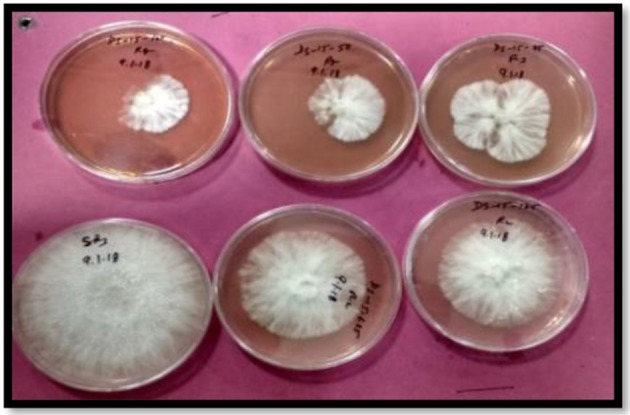
Fungicidal activity against *S. rolfsii* of *(2E)-1-ferrocenyl-3-(2,6-dichlorophenyl)-prop-2-en-1-one* **(3o)**.

**Figure 2:**
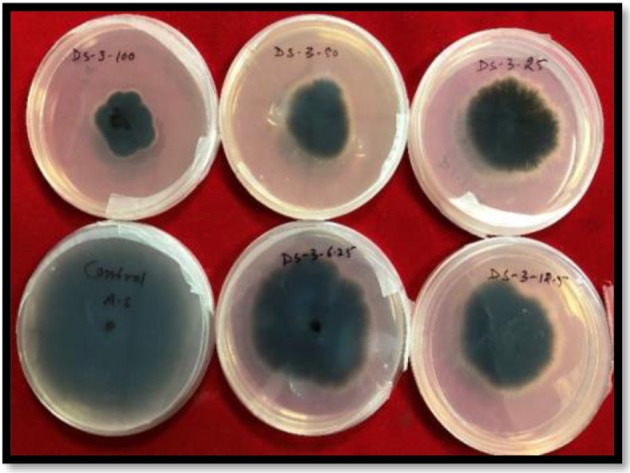
Fungicidal activity against *A. solani* of *(2E)-1-ferrocenyl-3-(4-fluorophenyl)-prop-2-en-1-one* **(3c)**.

All the synthesized compounds showed antifungal activity (Tables 2A, 3A). The compound **3o** i.e., (2E)-1-ferrocenyl-3-(2,6-dichlorophenyl)-prop-2-en-1-one was found to be most active against *S. rolfsii*, having ED_50_ value 23.24 mg L^−1^ as compared to commercial fungicide Hexaconazole 5% SC (ED_50_ = 8.5 mg L^−1^) (Figure 1). But in case of *A. solani*, compound **3c** i.e., (2E)-1-ferrocenyl-3-(4-fluorophenyl)-prop-2-en-1-one, having value ED_50_ = 29.9 mg L^−1^ showed highest activity (Figure 2). The corresponding value of commercial fungicide Mancozeb 75% WP was 2.9 mg L^−1^.

**Table 2A:** Experimental and predicted fungicidal activity of ferrocenyl chalcones against *S. rolfsii*.

**Test compound**	**Regression equation**	**χ^2^**	**ED_**50**_ (ppm)^a^**	**Fiducial limit**	**Experimental _P_**$\text{E}{\text{D}}_{50}^{\text{b}}$	**Predicted _P_ED_50_**		
						**PLS**	**MLR**	**PCR**
3a	0.56 × ± 0.82	0.671	32.30	19.24–64.67	**–**1.509	**–**1.595	**–**1.635	**–**1.577
3b	0.64 × ± 0.78	1.024	24.21	12.5–41.06	**–**1.366	**–**1.377	**–**1.380	**–**1.525
3c	0.56 × ± 0.82	0.053	38.11	22.33–91.72	**–**1.581	**–**1.548	**–**1.585	**–**1.528
3d	0.48 × ± 0.76	0.010	51.24	27.60–240.81	**–**1.710	**–**1.728	**–**1.702	**–**1.660
3e	0.40 × ± 0.70	0.233	68.29	34.40-128.56	**–**1.834	**–**1.630	**–**1.546	**–**1.660
3f	0.64 × ± 1.08	0.336	72.39	41.22-173.83	**–**1.860	**–**1.879	**–**1.867	**–**1.785
3g	0.4 × ± 1.2	0.017	650.32	425.12–1365.78	**–**2.813	**–**2.803	**–**2.816	**–**2.773
3h	0.4 × ± 0.8	0.232	214.29	68.15–425.14	**–**2.331	**–**2.415	**–**2.324	**–**2.449
3i	0.64 × ± 1.18	0.771	94.33	54.21–221.15	**–**1.975	**–**1.053	**–**1.425	**–**1.364
3j	0.4 × ± 0.9	0.202	188.81	67.45–325.15	**–**2.276	**–**2.240	**–**2.285	**–**2.217
3k	0.56 × ± 1.22	0.669	402.84	123.05–719.45	**–**2.605	**–**2.536	**–**2.612	**–**2.619
3l	0.4 × ± 0.9	0.140	213.47	70.50–456.98	**–**2.329	**–**2.540	**–**2.543	**–**2.554
3m	0.4 × ± 0.9	0.366	330.00	87.10–687.98	**–**2.519	**–**2.548	**–**2.512	**–**2.516
3n	0.48 × ± 0.76	0.144	40.32	20.04–96.41	**–**1.606	**–**1.606	**–**1.596	**–**1.608
3o	0.8 × ± 1	0.250	23.24	14.02–33.63	**–**1.384	**–**2.288	**–**1.970	**–**1.931
3p	0.56 × ± 0.82	0.155	27.76	16.47–49.25	**–**1.443	**–**1.060	**–**1.391	**–**1.323

a *Measured in vitro fungicidal activity against S. rolfsii*,

*^b^The negative logarithm of the measured ED_50_ (ppm), Hexaconazole 5% SC, ED_50_ = 8.57 ppm*.

**Table 2B:** Unicolumn statistics of training and test sets for fungicidal activity against *S. rolfsii*.

**Set**	**Average**	**Max**	**Min**	**Std. dev**.	**Sum**
Training	**–**2.0680	**–**1.3700	**–**2.8100	0.5021	**–**20.6800
Test	**–**1.7450	**–**1.3800	**–**2.3300	0.4381	**–**6.9800

**Table 2C:** Statistical results of 2D-QSAR models against *S. rolfsii*.

**Statistical parameters**	**Model-1 (PLS)**	**Model-2 (MLR)**	**Model-3 (PCR)**
n	11_Training_ 5_Test_	11_Training_ 5_Test_	11_Training_ 5_Test_
DF	7	8	7
r^2^	0.9925	0.9997	0.9760
q^2^	0.8704	0.9962	0.7736
*F* test	465.9636	3036.5304	142.4344
r^2^ se	0.0492	0.0122	0.0882
q^2^se	0.2049	0.0464	0.2709
pred_r^2^	0.0638	0.6201	0.6011
pred_q^2^	0.5934	0.3985	0.3634

**Table 3A:** Experimental and predicted fungicidal activity of ferrocenyl chalcones against *A. solani*.

**Test compound**	**Regression equation**	**χ^2^**	**ED_**50**_ (ppm)^a^**	**Fiducial limit**	**Experimental _P_**$\text{E}{\text{D}}_{50}^{\text{b}}$	**Predicted _P_ED_50_**		
						**PLS**	**MLR**	**PCR**
3a	0.64 × ± 0.98	0.479	42.44	27.42–84.75	**–**1.628	**–**1.629	**–**1.570	**–**1.560
3b	0.48 × ± 0.76	0.371	48.29	27.40–102.52	**–**1.684	**–**1.751	**–**1.781	**–**1.676
3c	0.56 × ± 0.82	0.226	29.90	16.21–65.34	**–**1.476	**–**1.479	**–**1.507	**–**1.647
3d	0.48 × ± 0.76	0.024	42.33	24.09–93.95	**–**1.627	**–**1.744	**–**1.759	**–**1.673
3e	0.56 × ± 0.82	0.389	45.14	25.71–105.87	**–**1.655	**–**1.792	**–**1.763	**–**1.766
3f	0.56 × ± 0.82	0.251	38.99	23.01–93.95	**–**1.591	**–**1.577	**–**1.694	**–**1.753
3g	0.64 × ± 1.08	0.336	72.40	41.22–173.78	**–**1.860	**–**1.752	**–**1.750	**–**1.808
3h	0.8 × ± 1.2	0.694	47.99	32.36–89.41	**–**1.681	**–**1.728	**–**1.711	**–**1.873
3i	0.4 × ± 0.7	0.217	71.13	36.10–150.87	**–**1.852	**–**1.776	**–**1.773	**–**1.583
3j	0.48 × ± 0.96	0.058	108.66	54.74–223.98	**–**2.036	**–**2.065	**–**2.063	**–**2.024
3k	0.48 × ± 0.96	0.088	185.40	67.92–369.12	**–**2.268	**–**2.254	**–**2.270	**–**2.228
3l	0.64 × ± 1.18	0.891	99.14	57.43–222.15	**–**1.996	**–**1.574	**–**1.506	**–**1.588
3m	0.56 × ± 0.82	0.522	35.36	20.81–78.43	**–**1.549	**–**1.573	**–**1.555	**–**1.737
3n	0.64 × ± 1.18	0.359	103.65	58.59–234.67	**–**2.016	**–**2.027	**–**1.975	**–**1.770
3o	0.8 × ± 1.2	0.516	48.32	33.10–86.82	**–**1.684	**–**1.909	**–**1.958	**–**1.782
3p	0.48 × ± 0.86	0.018	63.19	33.09–125.53	**–**1.801	**–**1.708	**–**1.800	**–**1.639

a *Measured in vitro fungicidal activity against A. solani*,

*^b^The negative logarithm of the measured ED_50_ (ppm), Mancozeb 75% WP ED_50_ = 2.96 ppm*.

**Table 3B:** Unicolumn statistics of training and test sets for fungicidal activity against *A. solani*.

**Set**	**Average**	**Max**	**Min**	**Std. dev**.	**Sum**
Training	**–**1.7960	**–**1.4800	**–**2.2700	0.2511	**–**17.9600
Test	**–**1.7800	**–**1.5900	**–**2.0000	0.1820	**–**7.1200

**Table 3C:** Statistical results of 2D-QSAR models against *A. solani*.

**Statistical parameters**	**Model-1(PLS)**	**Model-2(MLR)**	**Model-3(PCR)**
n	11_Training_ 5_Test_	11_Training_ 5_Test_	11_Training_ 5_Test_
DF	9	7	8
r^2^	0.9348	0.9357	0.6468
q^2^	0.7499	0.5369	0.1264
*F* test	50.2052	18.1880	14.6525
r^2^ se	0.0727	0.0854	0.1583
q^2^se	0.2917	0.4440	0.2489
pred_r^2^	0.4856	0.3644	0.2626
pred_q^2^	0.2826	0.3356	0.3041

**Figure 3:**
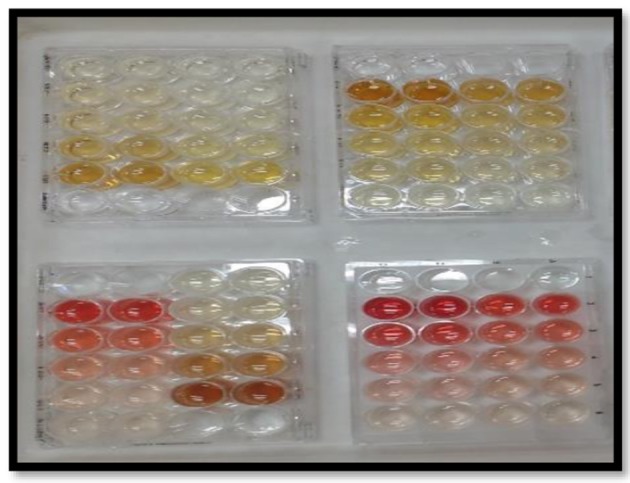
*In-vitro* nematicidal bioassay well culture plate containing nematode suspension (1 mL) and different concentrations (1 mL) of test compounds.

#### Nematicidal Activity

Although all the synthesized compounds possessed nematicidal activity against *M. incognita* (Table 4A), but the trend of nematicidal bioassay revealed that the compound *(2E)*-1-ferrocenyl- 3-(4-bromophenyl)-prop-2-en-1-one (**3b**) was more potent with LC_50_ values of 10.67 ppm as compared to commercial nematicide Carbofuran 3G (LC_50_ = 4.78 ppm) and Velum Prime 500 SC (LC_50_ = 2.40 ppm). It was found that mortality was directly proportional to time of exposure and concentration.

**Table 4A:** Experimental and predicted nematicidal activity of ferrocenyl chalcones against *M. incognita*.

**Test compound**	**Regression equation**	**χ^2^**	**LC_**50**_ (ppm)^a^**	**Fiducial limit**	**Experimental _P_**$\text{L}{\text{C}}_{50}^{\text{b}}$	**Predicted _P_LC_50_**		
						**PLS**	**MLR**	**PCR**
3a	0.8571 × ± 1.1429	0.220	25.81	19.23–40.39	**–**1.412	**–**1.410	**–**1.408	**–**1.298
3b	0.8929 × ± 0.8571	0.444	10.67	7.32–14.41	**–**1.028	**–**1.067	**–**1.067	**–**1.086
3c	0.8571 × ± 0.9429	0.414	20.16	14.54–29.69	**–**1.304	**–**1.318	**–**1.292	**–**1.277
3d	0.8571 × ± 0.9429	0.398	16.99	12.18–24.16	**–**1.230	**–**1.266	**–**1.269	**–**1.215
3e	0.8571 × ± 0.9429	0.571	14.58	10.28–20.37	**–**1.164	**–**1.097	**–**1.104	**–**1.086
3f	1 × ± 0.99	1.515	13.08	9.14–18.48	**–**1.117	**–**1.336	**–**1.274	**–**1.277
3g	0.8571 × ± 0.9429	0.198	19.41	14.09–28.03	**–**1.288	**–**1.303	**–**1.285	**–**1.277
3h	0.8571 × ± 0.9429	0.139	13.83	10.11–18.51	**–**1.141	**–**1.143	**–**1.141	**–**1.198
3i	0.8571 × ± 1.1429	0.571	25.95	18.93–39.63	**–**1.414	**–**1.383	**–**1.409	**–**1.388
3j	0.8571 × ± 1.1429	0.546	28.12	20.45–43.75	**–**1.449	**–**1.457	**–**1.439	**–**1.475
3k	0.8571 × ± 0.9429	0.41	22.32	16.13–33.38	**–**1.349	**–**1.344	**–**1.346	**–**1.277
3l	0.8929 × ± 1.1071	1.042	17.81	12.98–25.11	**–**1.251	**–**1.247	**–**1.228	**–**1.277
3m	0.8929 × ± 0.8571	0.261	11.04	7.66–14.88	**–**1.043	**–**1.284	**–**1.273	**–**1.277
3n	0.8571 × ± 0.9429	0.434	19.34	14.09–27.80	**–**1.286	**–**1.082	**–**1.080	**–**1.135
3o	0.8929 × ± 0.8571	0.58	12.66	8.91–17.23	**–**1.102	**–**1.112	**–**1.117	**–**1.135
3p	0.7143 × ± 0.8857	0.293	23.27	16.95–34.89	**–**1.367	**–**1.365	**–**1.386	**–**1.386

a *Measured in vitro nematicidal activity against M. incognita*,

*^b^The negative logarithm of the measured LC_50_ (ppm), Carbofuran 3G LC_50_ = 4.78 ppm, Velum Prime 500 SC LC_50_ = 2.40 ppm*.

**Table 4B:** Unicolumn statistics of training and test sets for nematicidal activity against *M. incognita*.

**Set**	**Average**	**Max**	**Min**	**Std. dev**.	**Sum**
Training	**–**1.2545	**–**1.0280	**–**1.4490	0.1355	**–**13.7990
Test	**–**1.2292	**–**1.0430	**–**1.4120	0.1478	**–**6.1460

**Table 4C:** Statistical results of 2D-QSAR models against *M. incognita*.

**Statistical parameters**	**Model-1 (PLS)**	**Model-2 (MLR)**	**Model-3 (PCR)**
n	11_Training_ 5_Test_	11_Training_ 5_Test_	11_Training_ 5_Test_
DF	8	6	8
r^2^	0.9531	0.9565	0.8784
q^2^	0.5037	**–**390.0000	0.7728
*F* test	81.3219	32.9806	78.9078
r^2^ se	0.0328	0.0365	0.0528
q^2^se	0.1067	**–**39052.1351	0.0722
pred_r^2^	0.4347	**–**0.3231	0.4818
pred_q^2^	0.1924	0.1731	0.1703

**Table 5:** Molecular descriptors used in QSAR study.

**Descriptor**	**Description**
NitrogensCount	This descriptor signifies number of nitrogen atoms in a compound
Most+ve&–ve Potential Distance	This descriptor signifies the distance between points having the highest value of +ve and highest value of –ve electrostatic potential on van der Waals surface area of the molecule
DeltaPsiA	A measure of hydrogen–bonding propensity of the molecules
T_T_C_4	Number of atoms which are separated from carbon atom by four bonds
T_T_C_3	Number of atoms which are separated from carbon atom by three bonds
T_N_O_6	Denotes number of single/multiple bonded Nitrogen in molecule which are six bonds away from any single/multiple bonded oxygen atom
chiV2	This descriptor signifies atomic valence connectivity index (order 2)
SKMostHydrophobicHydrophilicDistance	This descriptor signifies distance between most hydrophobic and hydrophilic point on the vdW surface
FluorinesCount	This descriptor signifies number of fluorine atoms in a compound
SaasCE-index	Electrotopological state indices for number of carbon atom connected with one single bond along with two aromatic bonds.
BalabanIndexJ	J = (E/μ+1) ∑ (dsi, dsj) Where dsi, dsj = sum of the row i and j of the distance matrix, E = number of edges, μ = Number of rings in a molecule
MomInertiaX	This descriptor signifies moment of interia at X-axix
DeltaAlphaA	A measure of count of non-hydrogen heteroatoms
SsOHE-index	Electrotopological state indices for number of –OH group connected with one single bond.
SaaCHcount	This descriptor defines the total number of carbon atoms connected with a hydrogen along with two aromatic bonds
Epsilon4	Measure of electronegative atom count including hydrogen atoms with respect to the saturated hydrocarbon (reference alkane) created from the molecule/fragment under consideration
Average-vePotential	This descriptor signifies the average of the total -ve electrostatic potential on van der Waals surface area of the molecule
ZcompDipole	This descriptor signifies the z component of the dipole moment (external coordinates).
k1alpha	This descriptor signifies first alpha modified shape index: s (s-1)^2^/m^2^ where s = n + a

#### 2D-QSAR Study

**Figure 4:**
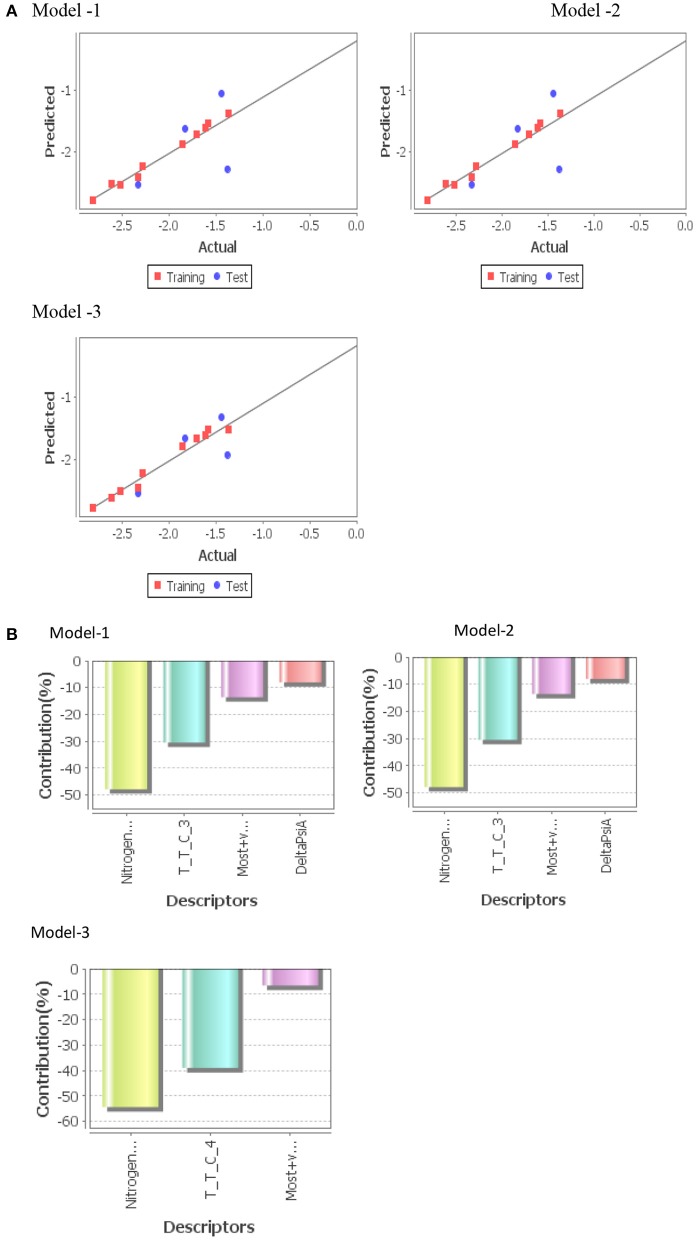
**(A)** Graphs of experimental vs. predicted fungicidal activity of different models against *S. rolfsii*. **(B)** Contribution charts of 2D-QSAR model.

**Figure 5:**
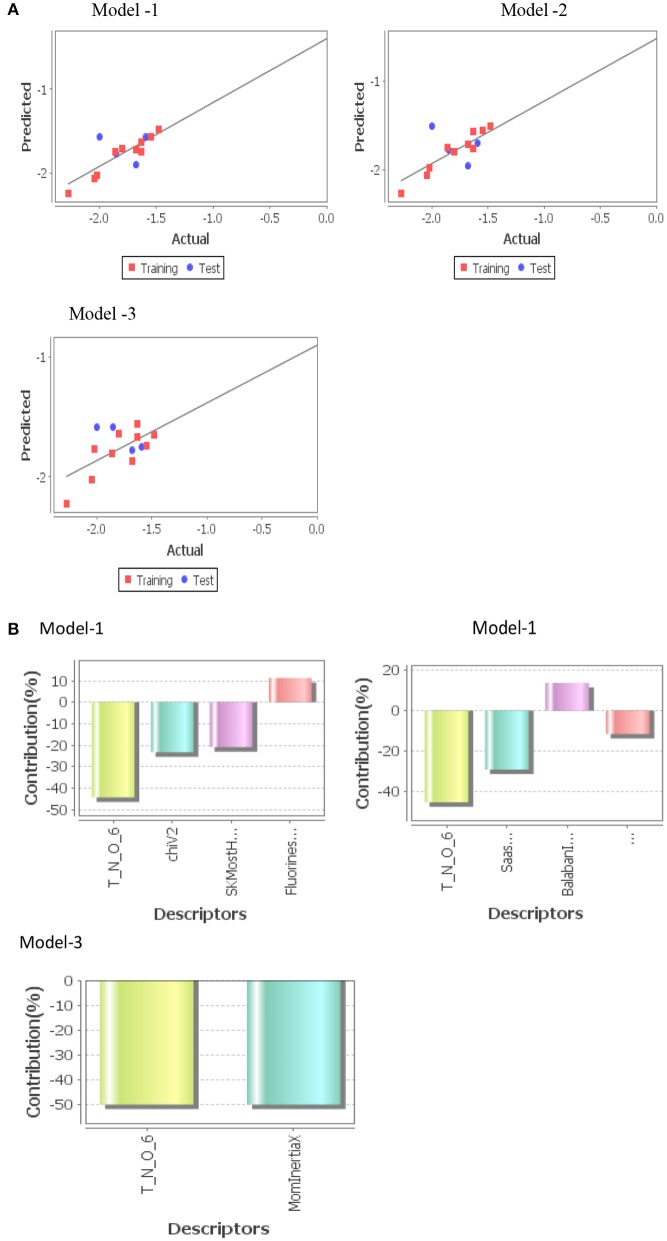
**(A)** Graphs of experimental vs. predicted fungicidal activity of different models against *A. solani*. **(B)** Contribution charts of 2D-QSAR models.

**Figure 6:**
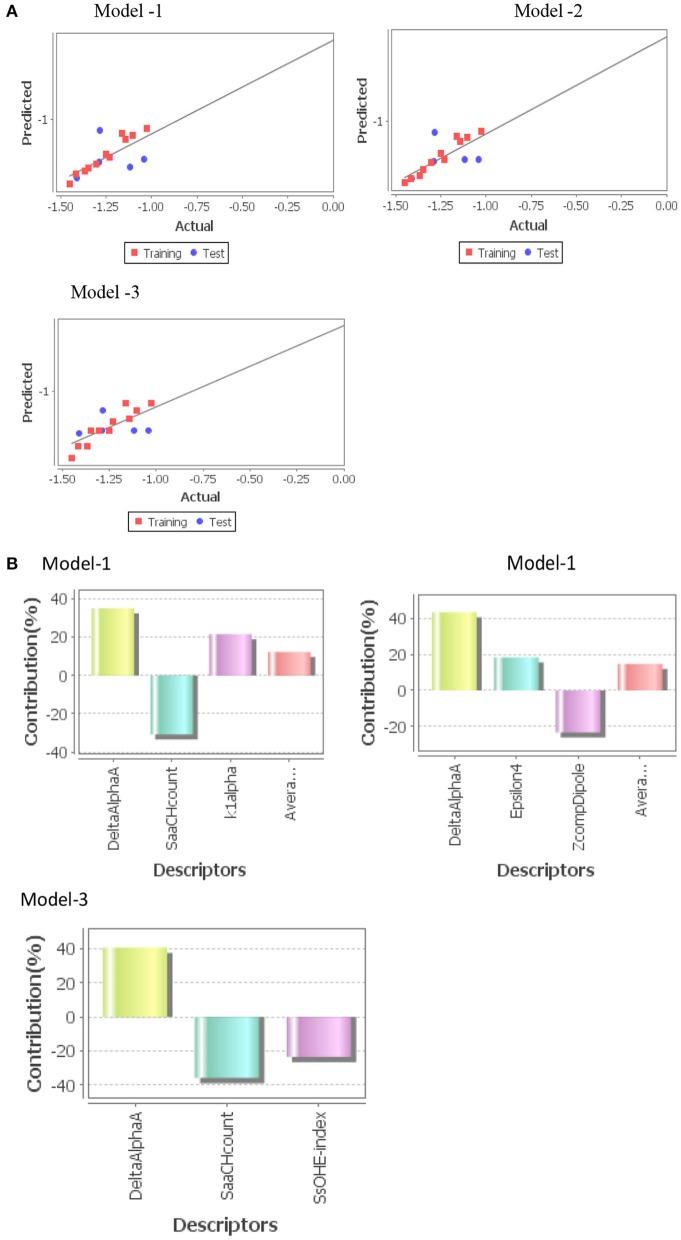
**(A)** Graphs of experimental vs. predicted nematicidal activity of different models against *M. incognita*. **(B)** Contribution charts of 2D-QSAR models.

Three statistically significant QSAR models *viz*. Model-1 (PLS), Model-2 (MLR), and Model-3 (PCR) were developed in 2D-QSAR analysis of fungicidal activity against *S. rolfsii* and *A. solani* and nematicidal activity against *M. incognita*. Fitness plot of each model was used for validation of statistical significance of model (Figures 4A, 5A, 6A).

***S. rolfsii***

Model-2 (MLR)

(1) $$\begin{array}{l} pED50=\;-\;0.8010\;\left(\text{NitrogensCount}\right)-\;0.0388\;\left(\text{T\_T\_C\_}3\right)\\ \;-\;0.3310\;\left(\text{Most}+\text{ve\&}-\text{vePotentail Distance}\right)\\ \;-\;1.6645\;\left(\text{DeltaPsiA}\right)+\;1.8574 \end{array}$$

Where n = 11, DF = 8, r^2^ = 0.9997, q^2^ = 0.9962, F_test = 3036.53, r^2^_se = 0.0122, q^2^_se = 0.0464, pred_r^2^ = 0.6201, pred_r^2^se = 0.3985

***A. solani***

Model-1 (PLS)

(2) $$\begin{array}{l} pED50=\;-\;0.6965\;\left(\text{T\_N\_O\_}6\right)-\;0.1945\;\left(\text{chiV}2\right)\\ \;-\;0.0764\text{ SKMostHydrophobicHydrophilic Distance}\\ \;+\;0.1774\text{ FluorinesCount }+\;0.3046 \end{array}$$

Where n = 11, DF = 9, r^2^ = 0.9348, q^2^ = 0.7499, F_test = 50.2052, r^2^_se = 0.0727, q^2^_se = 0.2917, pred_r^2^ = 0.4856, pred_r^2^se = 0.2826

***M. incognita***

Model-3(PCR)

(3) $$\begin{array}{l} pED50=\;+\;4.5874\left(\text{ DeltaAlphaA}\right)\;-\;0.0396\left(\text{ SaaCHcount}\right)\\ \;-\;0.0118\left(\text{ SsOHEindex}\right)\;-1.1185\; \end{array}$$

Where n = 11, DF = 8, r^2^ = 0.8784, q^2^ = 0.7728, F_test = 78.9078, r^2^_se = 0.0528, q^2^_se = 0.0722, pred_r^2^ = 0.4818, pred_r^2^se = 0.1703.

The biological activity variance for all QSAR models was estimated by multiplying the correlation coefficient (r^2^) with 100. The predictive power (q^2^) of models was determined by LOO (Left Out One) method. F denotes ratio of variance of models and that of error in regression. Models with higher F and lower SE of estimation *viz*. r^2^se and q^2^se were found statistically significant. External validation with pred_r^2^ > 0.3 confirmed the predictive power of QSAR model. The best model was determined by r^2^, q^2^, higher values of pred_r^2^ *F*_test and (Tables 2C, 3C, 4C). The best measure of authenticity of 2D QSAR is higher q^2^ value, because overfitting to data may result into higher r^2^ value. Mostly, a value of q^2^ >0.5 is found acceptable (Golbraikh and Tropsha, 2002; Doweyko, 2004; Ponce et al., 2004).

Alignment Independent (AI) descriptors were calculated as explained in Baumann's paper based on type of atom, bond and molecular topology (Balaban, 1982). Maximum three and minimum one attribute was given for every atom. First attribute “T” denotes molecule topology. Second attribute was denoted by atomic symbol and third by atoms with multiple (double or triple) bonds. Then selective distance count statistics, which counts all the fragments between first and last atom separated by graph distance. Graph distance is the minimum number of atoms along the path connecting two atoms in molecular structure. Topological indices are numerical values about chemical compositions which determines correlation between chemical structure and biological activity. AI descriptors were calculated by using attributes *viz*. 2 (atom with double bond), 3(atom with double bond), C (Carbon), N (Nitrogen), O (Oxygen), S (Sulfur), H (Hydrogen), F (Flourine), Cl (Chlorine), and Br (Bromine) with distance ranging from 0 to 7.

***Model-2 (MLR)*** was found to be the best model for QSAR study molecules against *S. rolfsii*. The developed model indicated that descriptors, Nitrogens Count, Most+ve&–vePotential Distance, DeltaPsiA, and AI descriptor, T_T_C_3 were inversely related to the fungicidal activity (Figure 4B). The major descriptor (~50%) is Nitrogen Count which negatively influences the fungicidal activity of test compounds. Most+ve&–vePotential Distance is an electrostatic descriptor which denotes distance between points having the highest value of +ve and highest value of –ve electrostatic potential on van der Waals surface area of the molecule. The result showed that it negatively influences the fungicidal activity of compounds. Therefore, lesser negative potential distance resulted in higher fungicidal activity.

***Model-1 (PLS)*** was found to be the best model for QSAR study molecules against *A. solani*.The developed model indicated that descriptors, T_N_O_6, chiV2, SKMostHydrophobic HydrophilicDistance were inversely related to the fungicidal activity (Figure 5B). The major descriptor (~50%) was T_N_O_6, which negatively influences the fungicidal activity of test compounds. But FluorinesCount positively influences the fungicidal activity of test compounds.

***Model-3 (PCR)*** was found to be the best model for QSAR study molecules against *M. incognita*. The developed model indicated that descriptors, DeltaAlphaA positively influences the nematicidal activity of test compounds and SaaCHcount & SsOHE-index were negatively influences the activity (Figure 6B). These two (DeltaPsiA & SaaCHcount) are major descriptors for nematicidal activity. DeltaAlphaA indicate that a greater number of non-hydrogen heteroatoms will be more nematicidal activity. SaaCHcount indicate that the total number of carbon atoms connected with a hydrogen along with two aromatic bonds will be less nematicidal activity.

## Conclusions

This is the first report of microwave assisted synthesis and bioefficacy (fungicidal and nematicidal) evaluations of ferrocenyl chalcones. Out of 16 synthesized compounds (2E)-1-ferrocenyl-3-(3-hydroxyphenyl)-prop-2-en-1-one (**3i**) is new molecule which was characterized by various spectroscopic techniques. The compound **3o** was found to be most active against *S. rolfsii* and the compound **3c** showed highest activity against *A. solani*. The compounds **3b, 3m, 3o, 3f**, and **3h** were most active against root-knot nematode *M. incognita*. QSAR studies was performed to find quantitative relationship between fungicidal and nematicidal activity and physicochemical/structural properties of the synthesized chalcones. The developed models were evaluated and validated for their external predictive power and statistical significance. Among the three 2D-QSAR models (MLR, PCR, and PLS), MLR, PLS, and PCR analysis showed significant predictive power and reliability for *S. rolfsii, A. solani*, and *M. incognita*, respectively. Information and understanding of descriptors affecting fungicidal and nematicidal activity of these ferrocenyl chalcones be used for structure optimization to attain higher activity.

## Data Availability Statement

All datasets generated for this study are included in the article/Supplementary Material.

## Author Contributions

NS: conceptualization, draft editing, and supervision. DY: methodology, data creation, analysis, and original draft writing. PK: methodology and analysis. P: methodology and nematicidal analysis. VR: draft editing and supervision. DKa, methodology and fungicidal analysis. DKh: analysis and funding.

### Conflict of Interest

The authors declare that the research was conducted in the absence of any commercial or financial relationships that could be construed as a potential conflict of interest.

## Supplementary Material

Supplementary Data Sheet 1 ^1^H-NMR, ^13^C-NMR, IR, and HR-MS spectra of compounds synthesized by Scheme 1.

Supplementary Data Sheet 2 ^1^H-NMR, ^13^C-NMR, IR, and HR-MS spectra of compound synthesized by Scheme 2.

Supplementary Data Sheet 3 Enlarge view of NMR spectra.

## Abbreviations

HR-MS: High Resolution-Mass Spectrometry
^1^H-NMR: Proton Nuclear Magnetic Resonance
^13^C-NMR: Carbon-13 Nuclear Magnetic Resonance
MM: Microwave Method
CM: Conventional Method
QSAR: Quantitative Structural Activity Relationship
TLC: Thin Layer Chromatography
TMS: Tetramethylsilane
PDA: Photo Diode Array
FT-IR: Fourier-transform infrared
ITCC: Indian Type Culture Collection
PDA: Potato Dextrose Agar
PLS: Partial Least Squares
MLR: Multiple Linear Regression
PCR: Principal Component Regression
UFLC: Ultra-Fast Liquid Chromatography.
