# Supplementary material for: Microwave Assisted Synthesis, Characterization and Biological Activities of Ferrocenyl Chalcones and Their QSAR Analysis
Source: Front Chem. 2019 Nov 26;7:814. doi: 10.3389/fchem.2019.00814 (PMC6901998; doi:10.3389/fchem.2019.00814)
Supplement: Supplementary Data Sheet 1 — 1H-NMR, 13C-NMR, IR, and HR-MS spectra of compounds synthesized by Scheme 1. [file Data_Sheet_1.PDF]

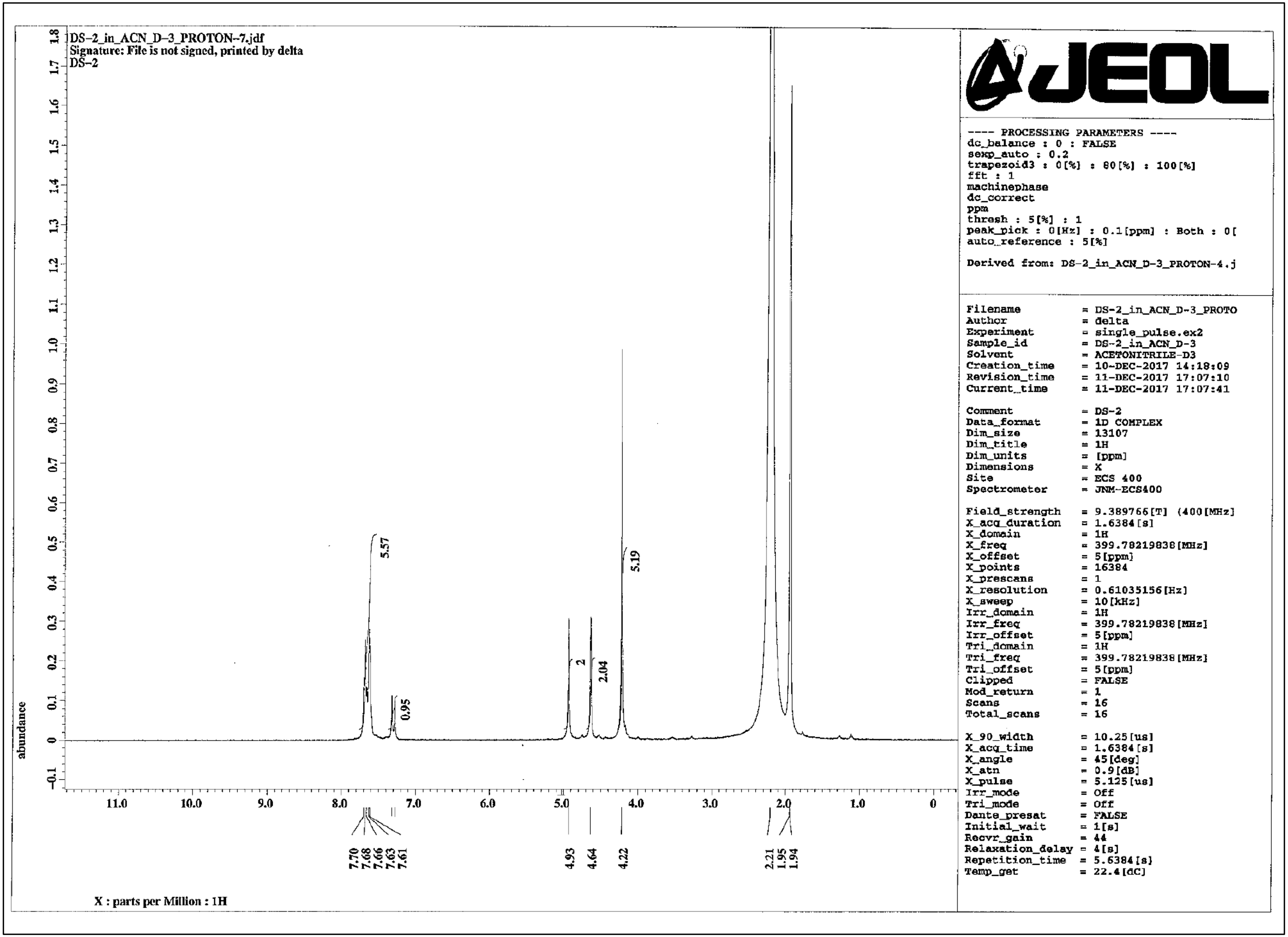

^{1}H NMR spectra of compound 3b

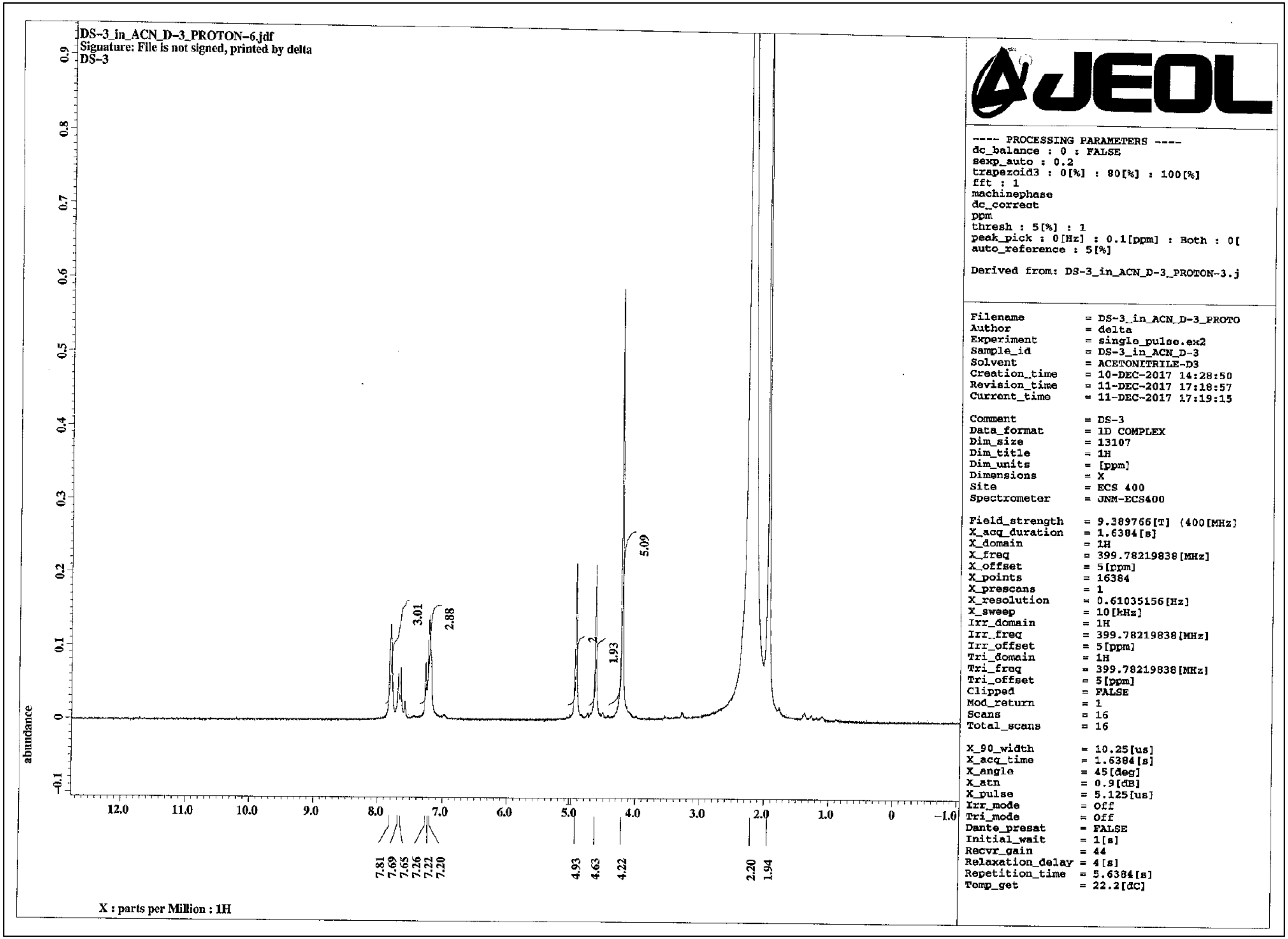

^{1}H NMR spectra of compound 3c

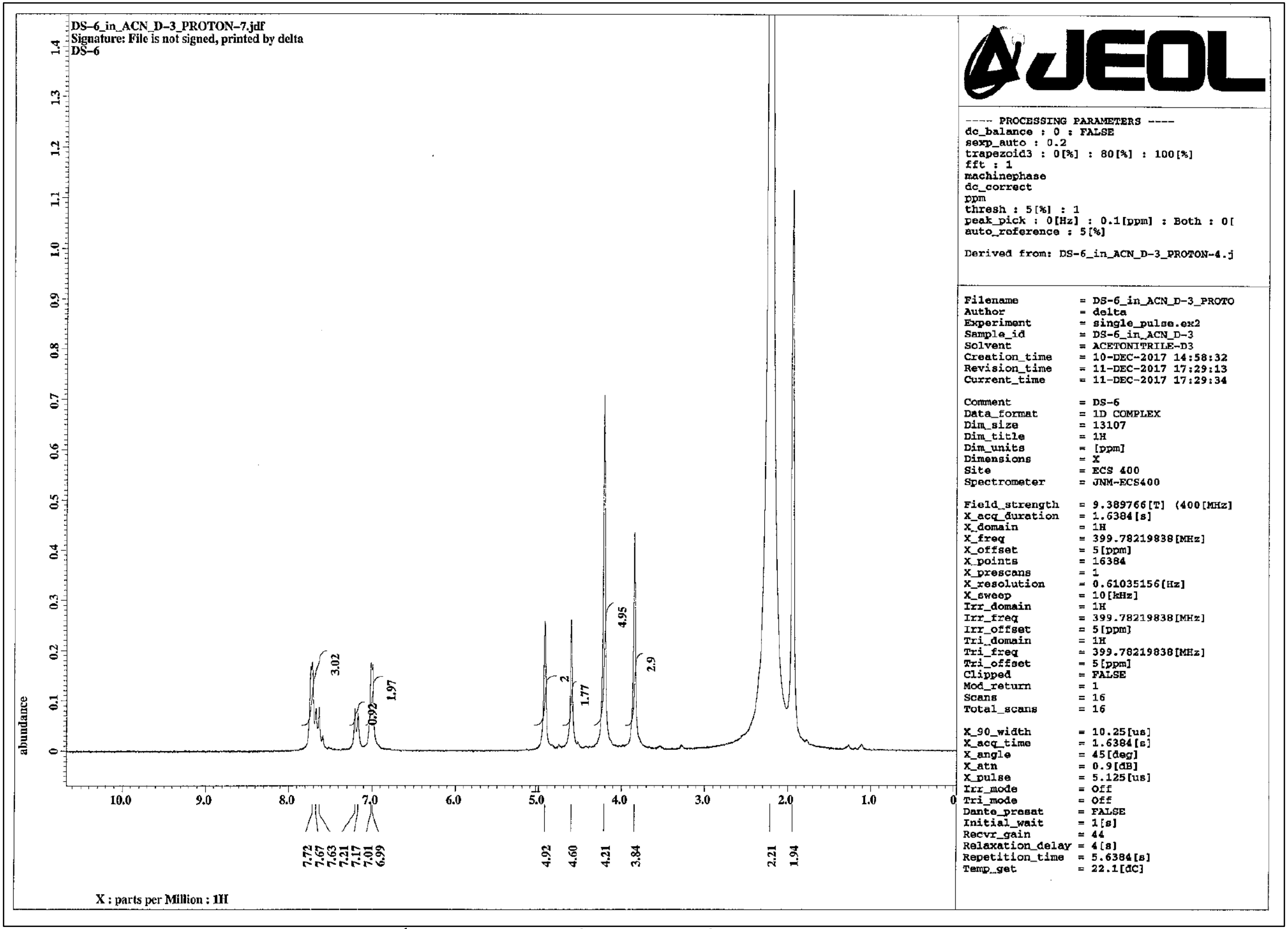

^{1}H NMR spectra of compound 3f

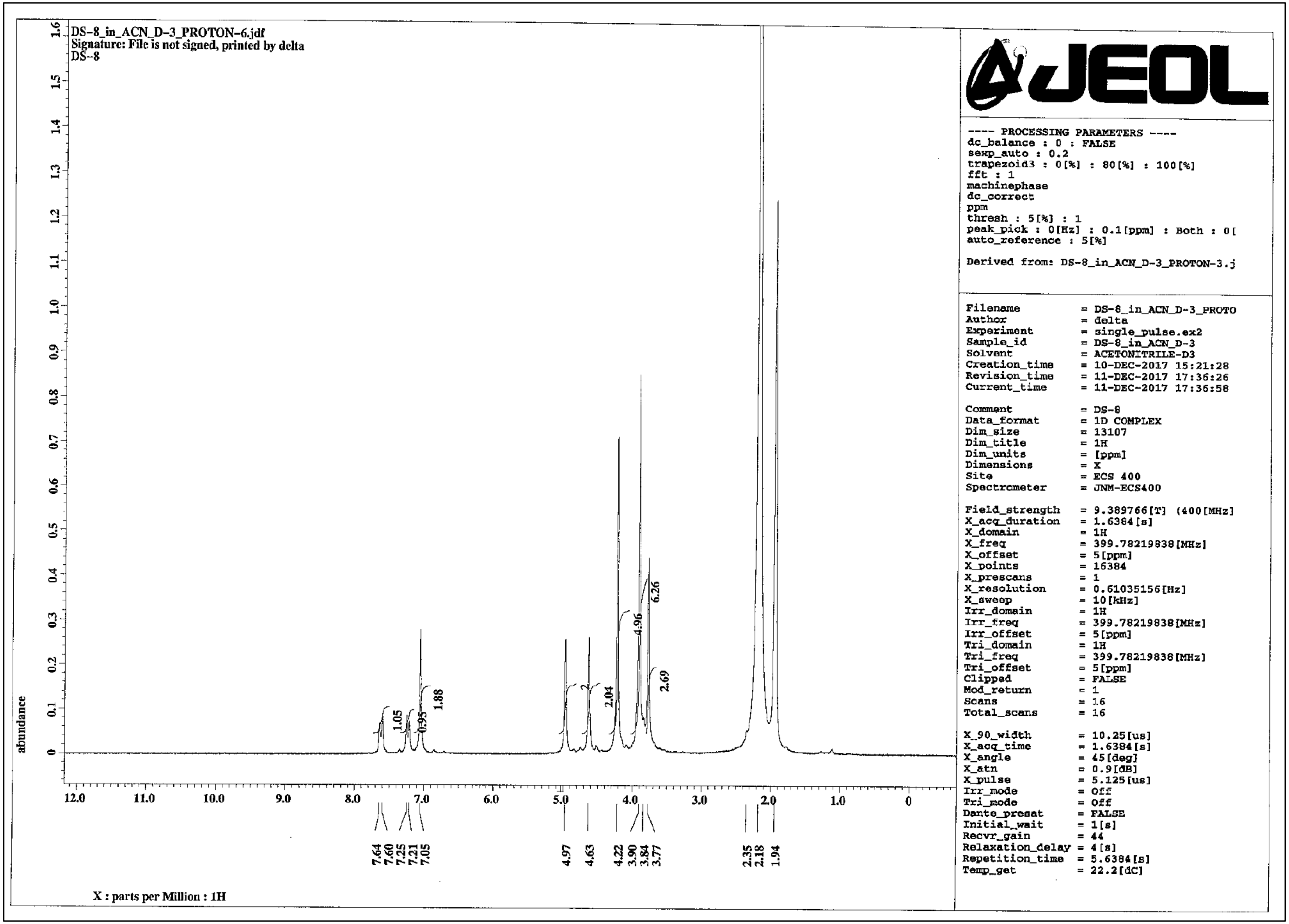

^{1}H NMR spectra of compound 3h

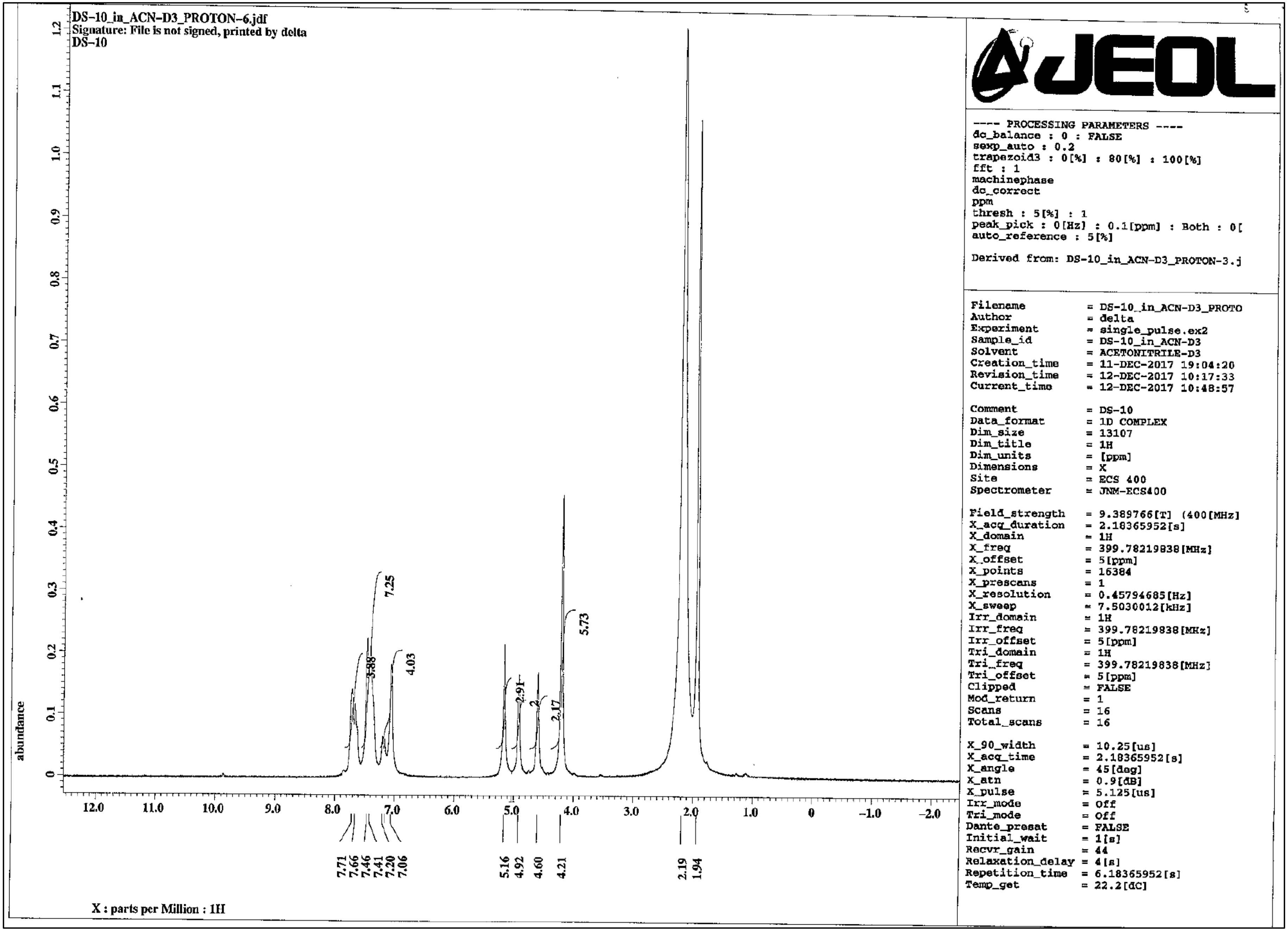

^{1}H NMR spectra of compound 3j

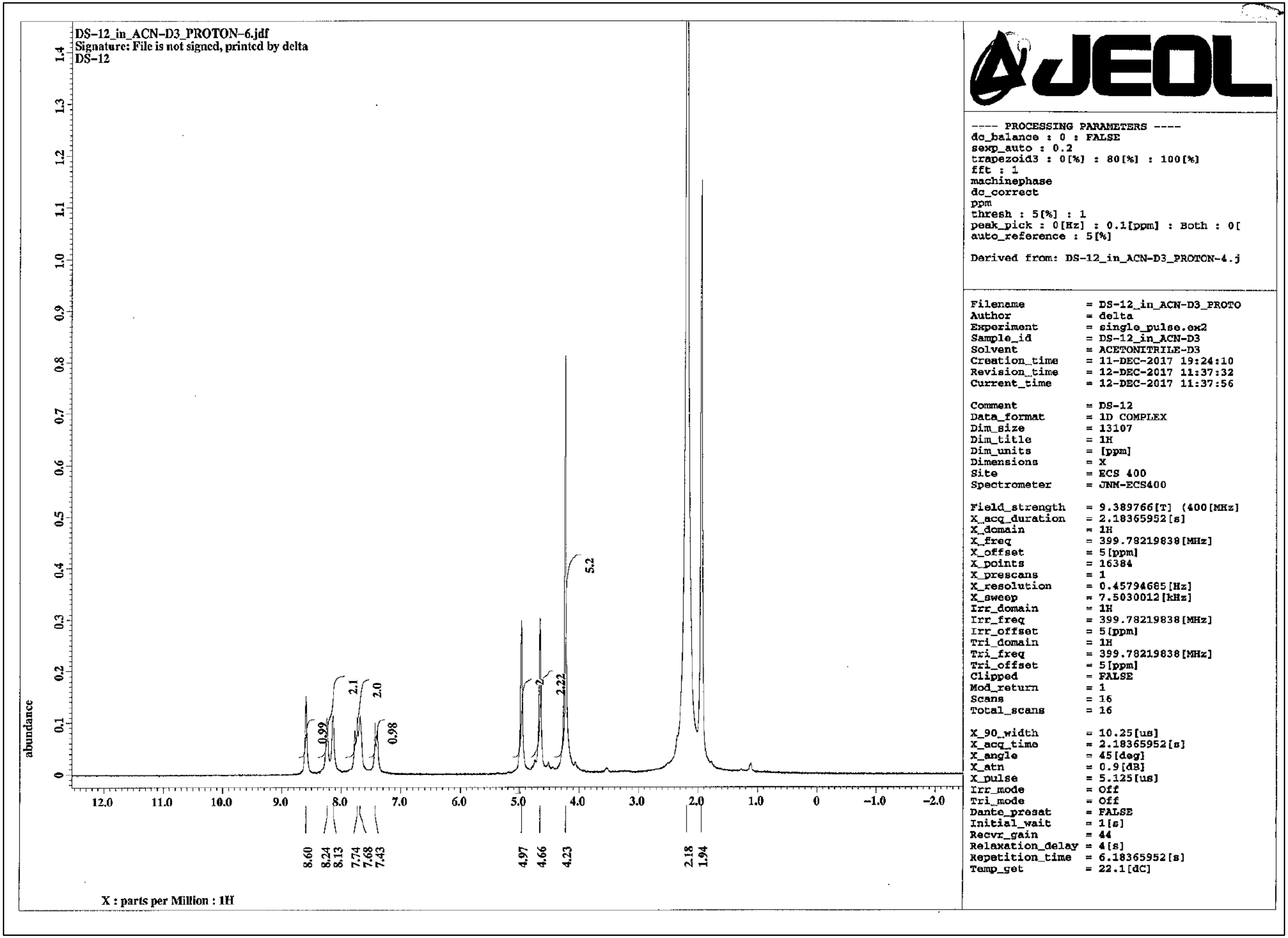

^{1}H NMR spectra of compound 3l

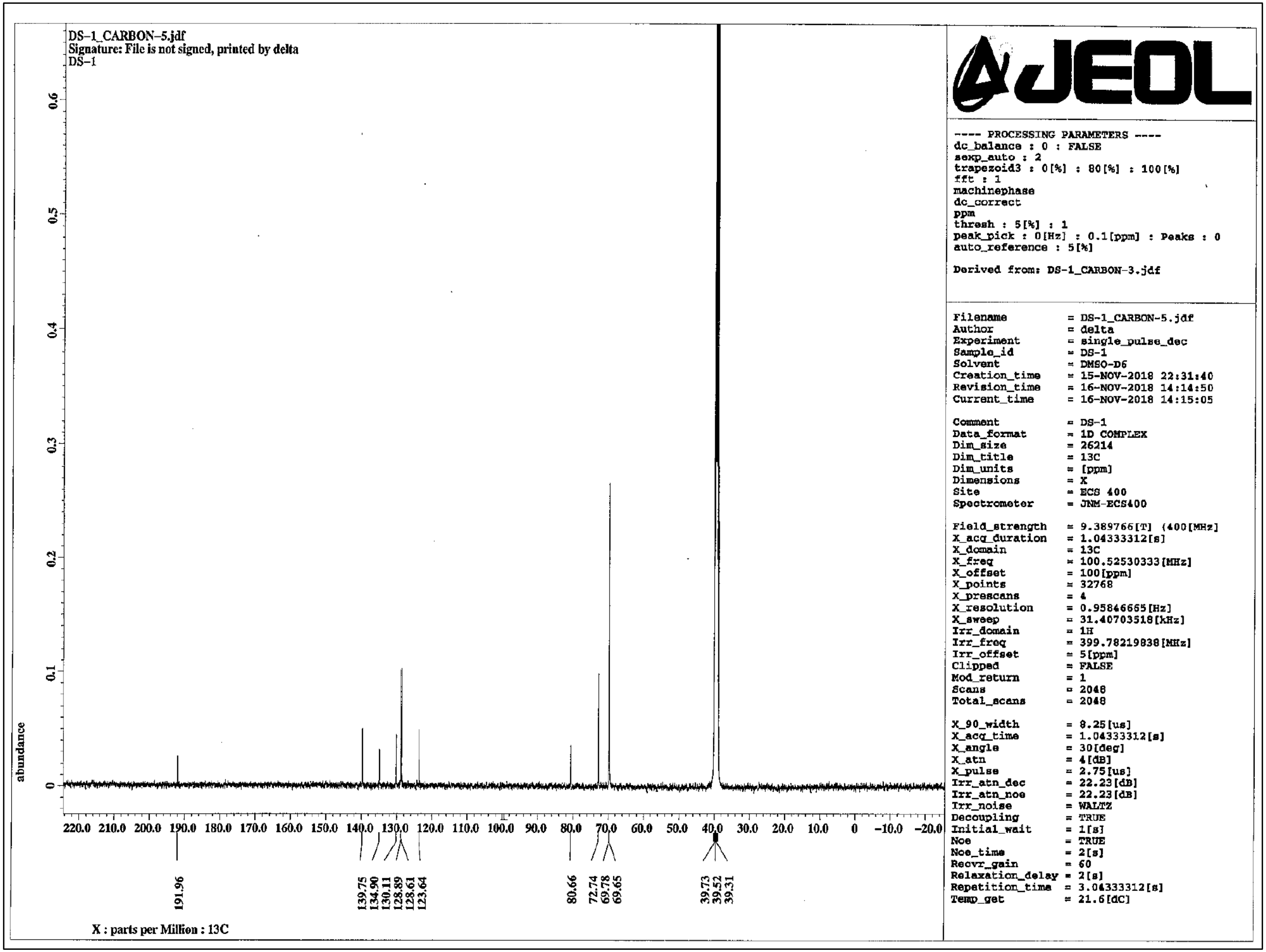

^{13}C NMR spectra of compound 3a

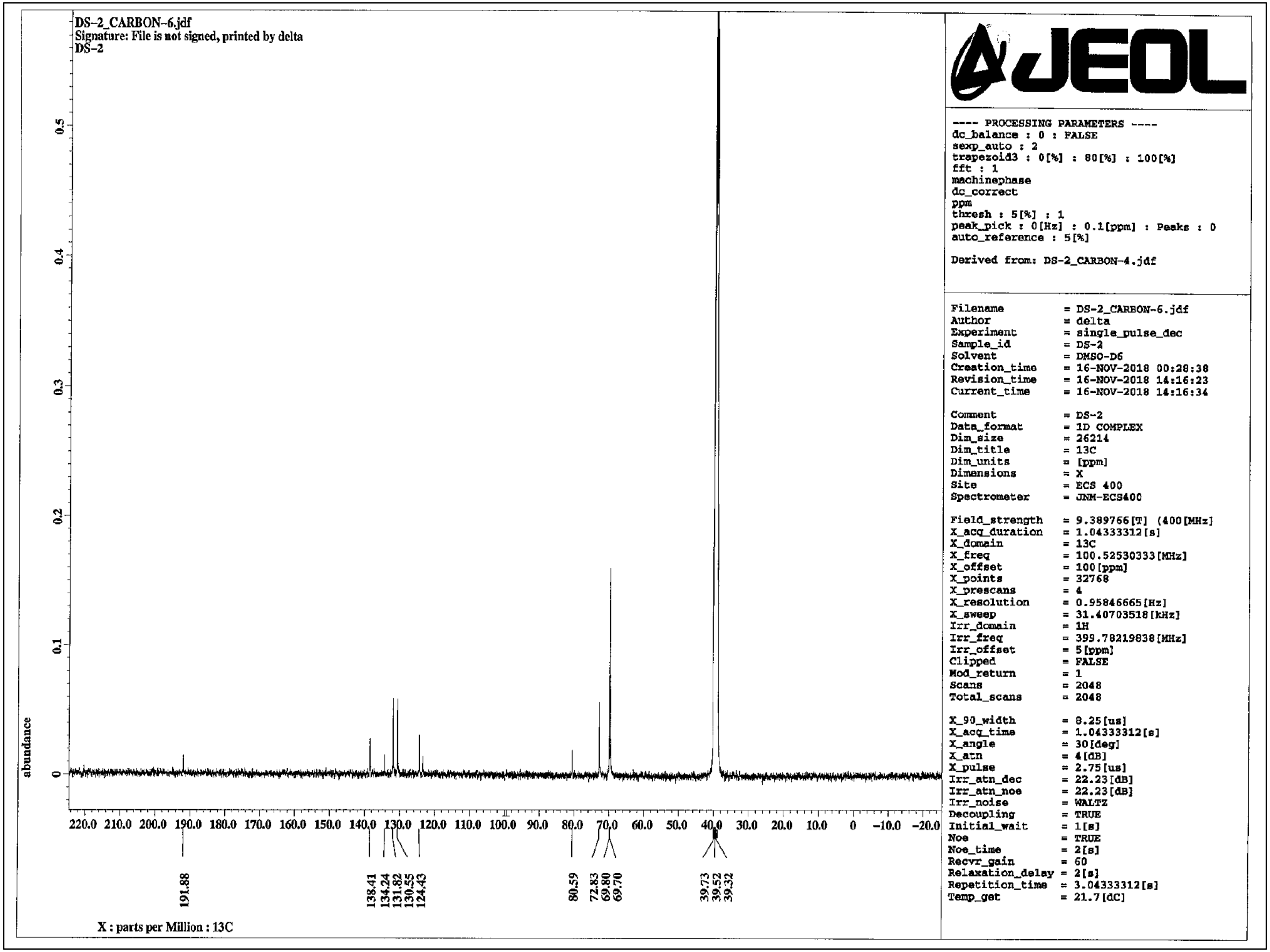

^{13}C NMR spectra of compound 3b

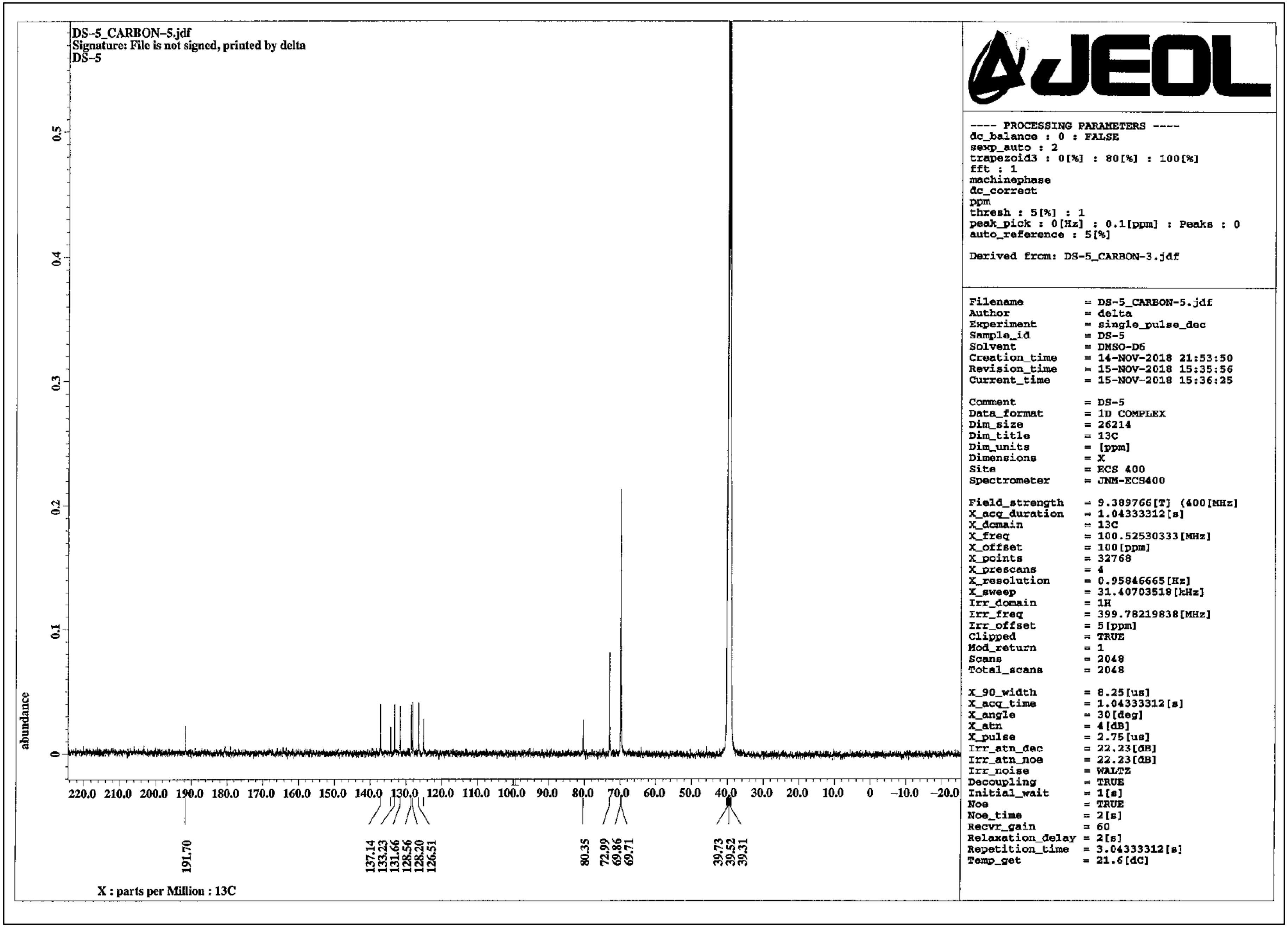

^{13}C NMR spectra of compound 3e

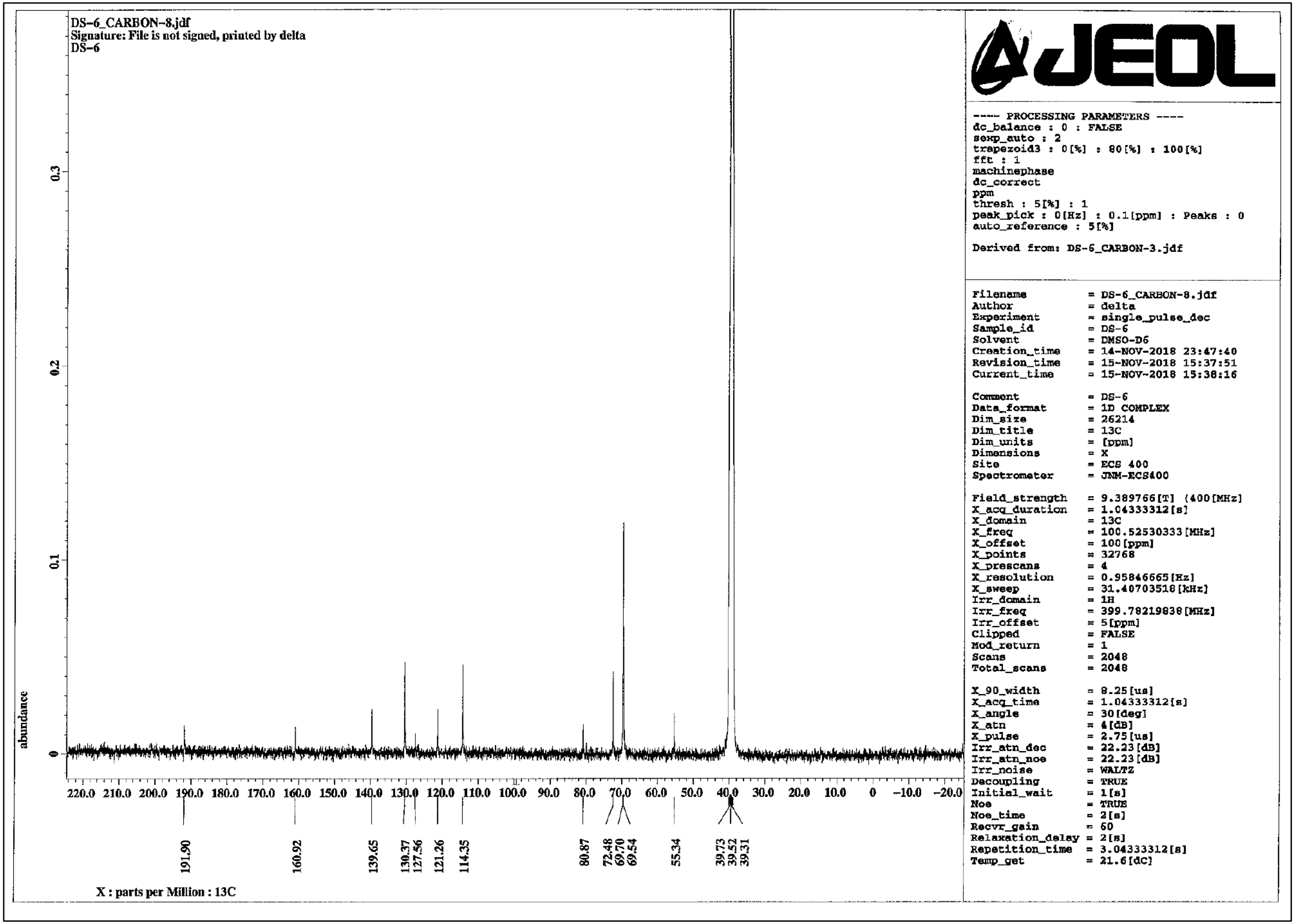

^{13}C NMR spectra of compound 3f

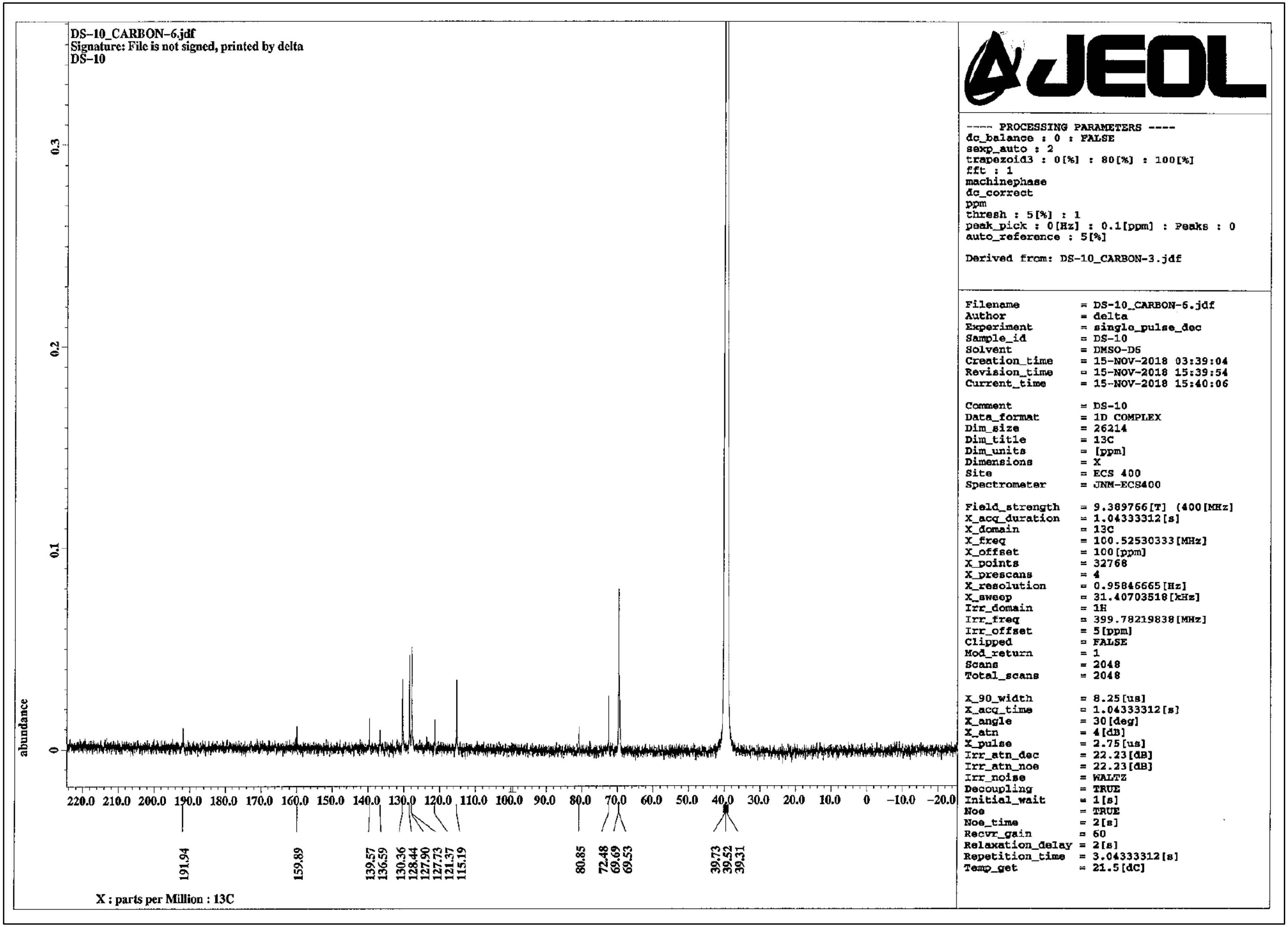

^{13}C NMR spectra of compound 3j

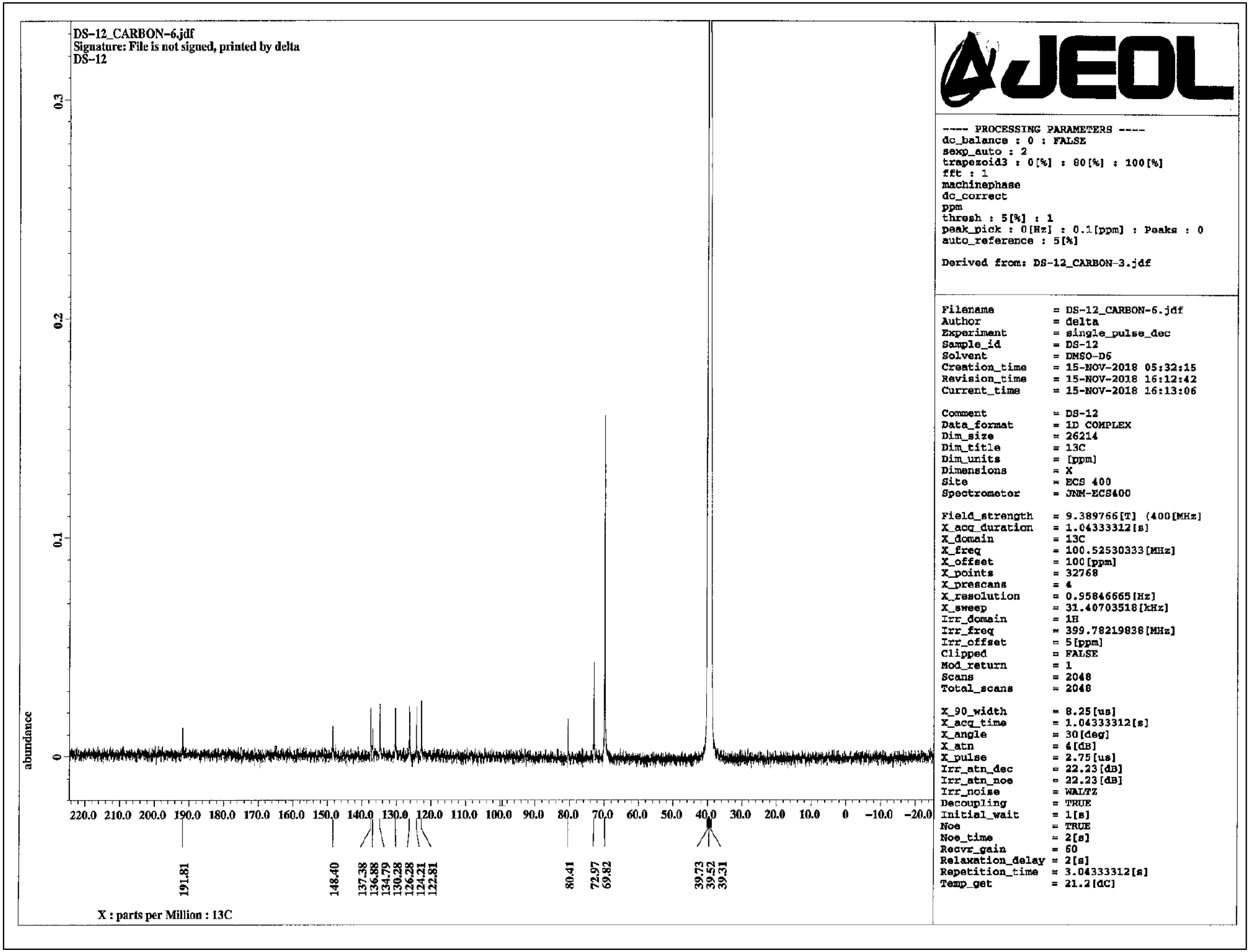

^{13}C NMR spectra of compound 3l

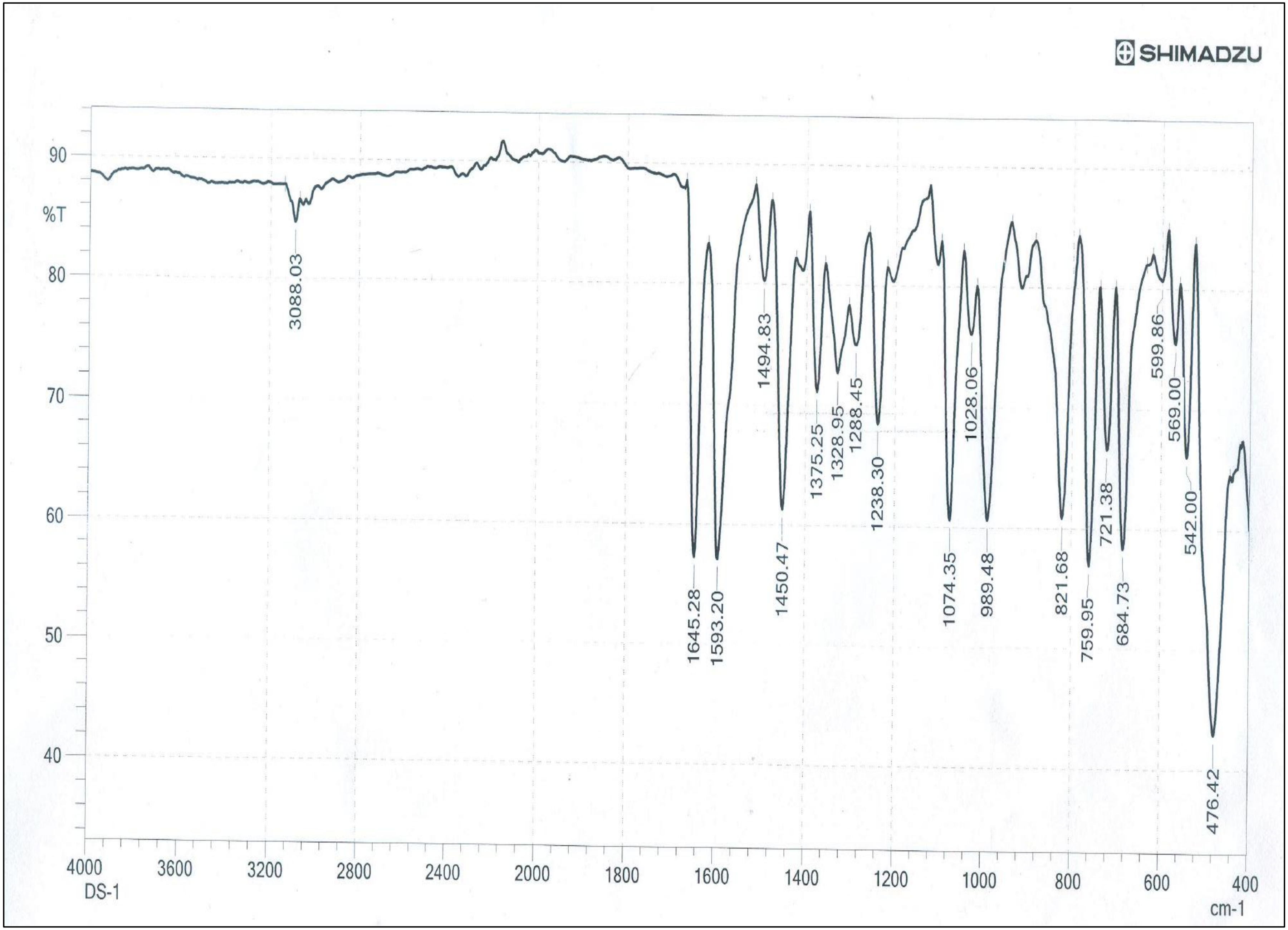

IR spectra of compound 3a

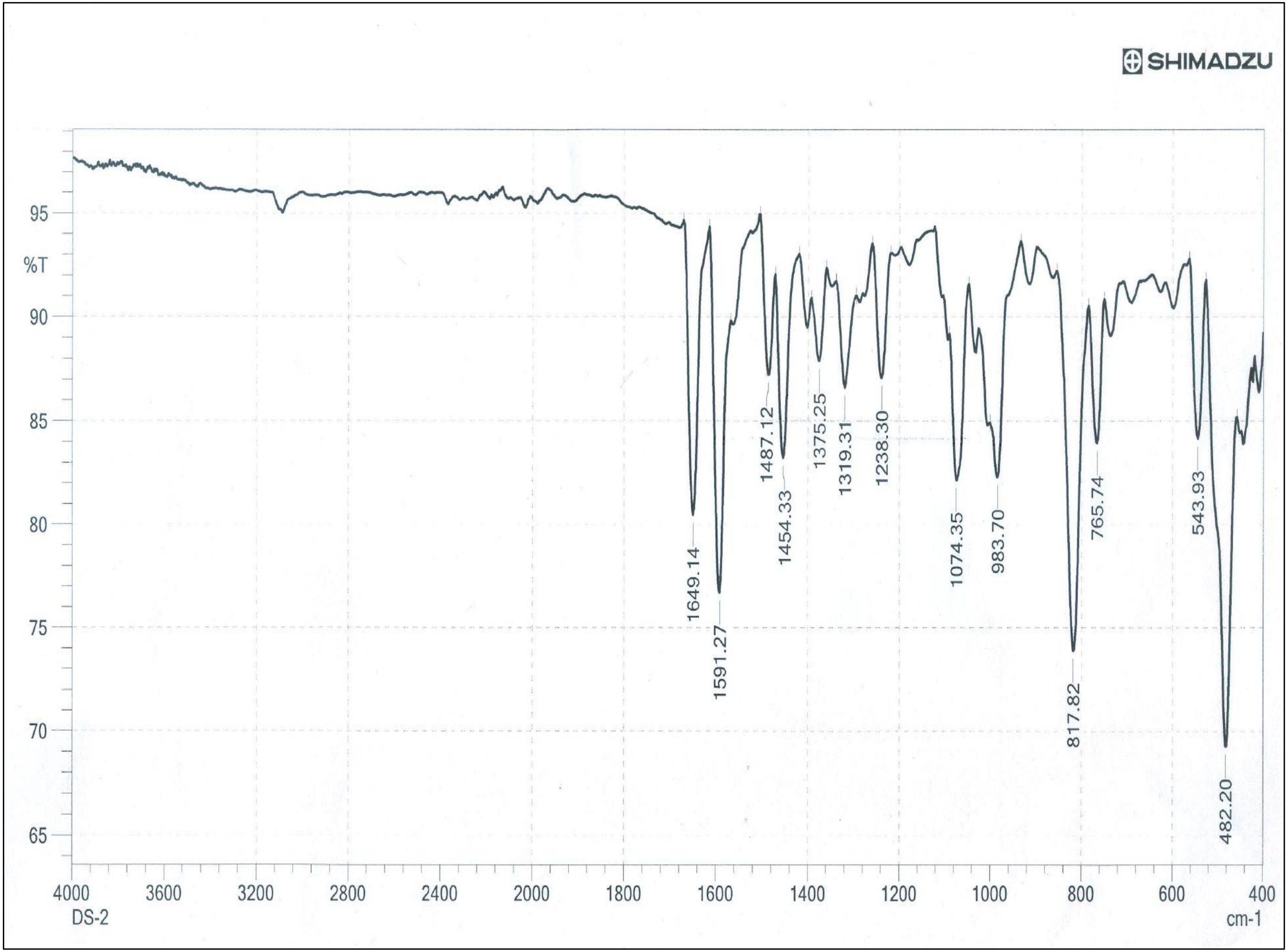

IR spectra of compound 3b

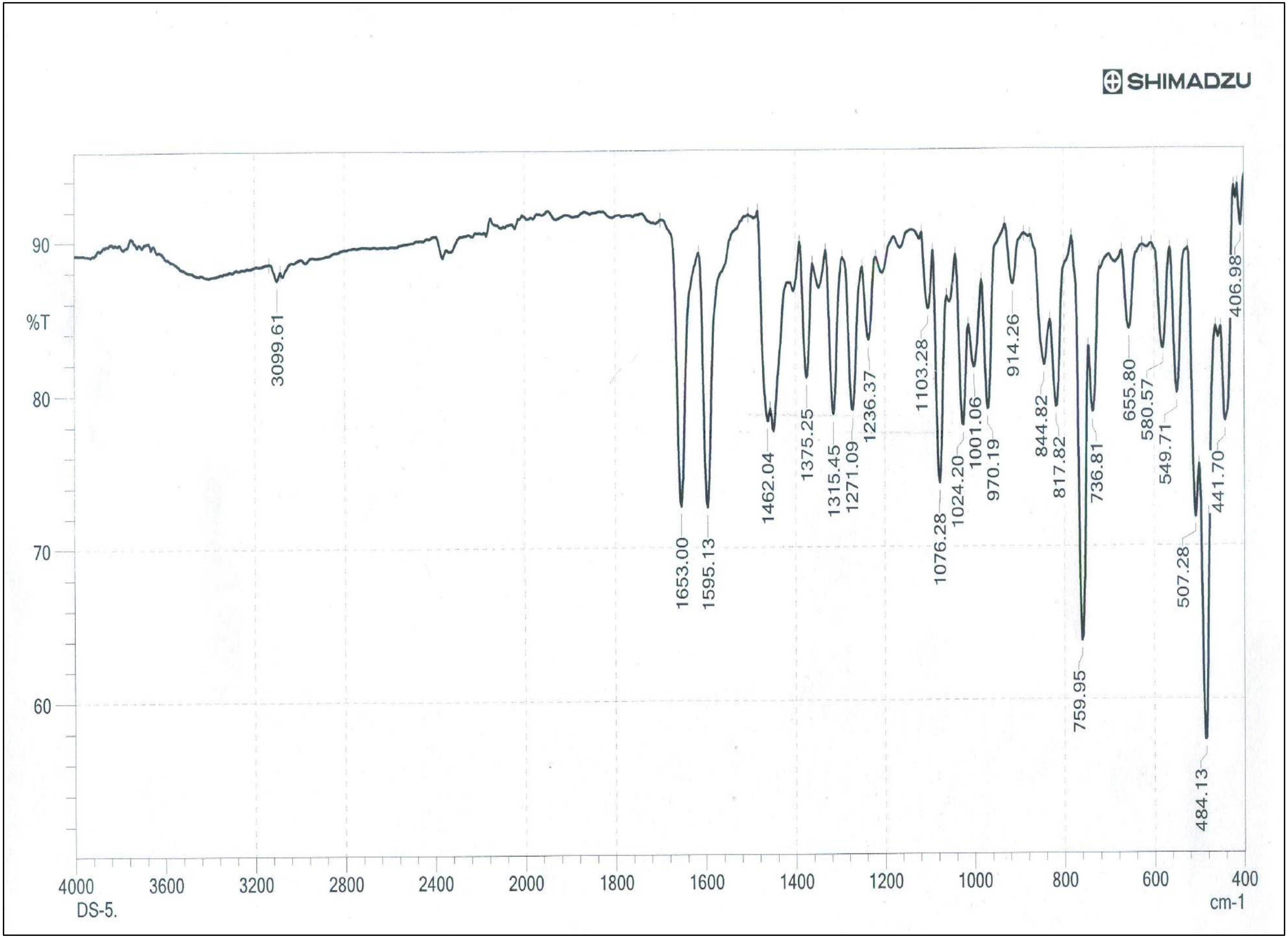

IR spectra of compound 3e

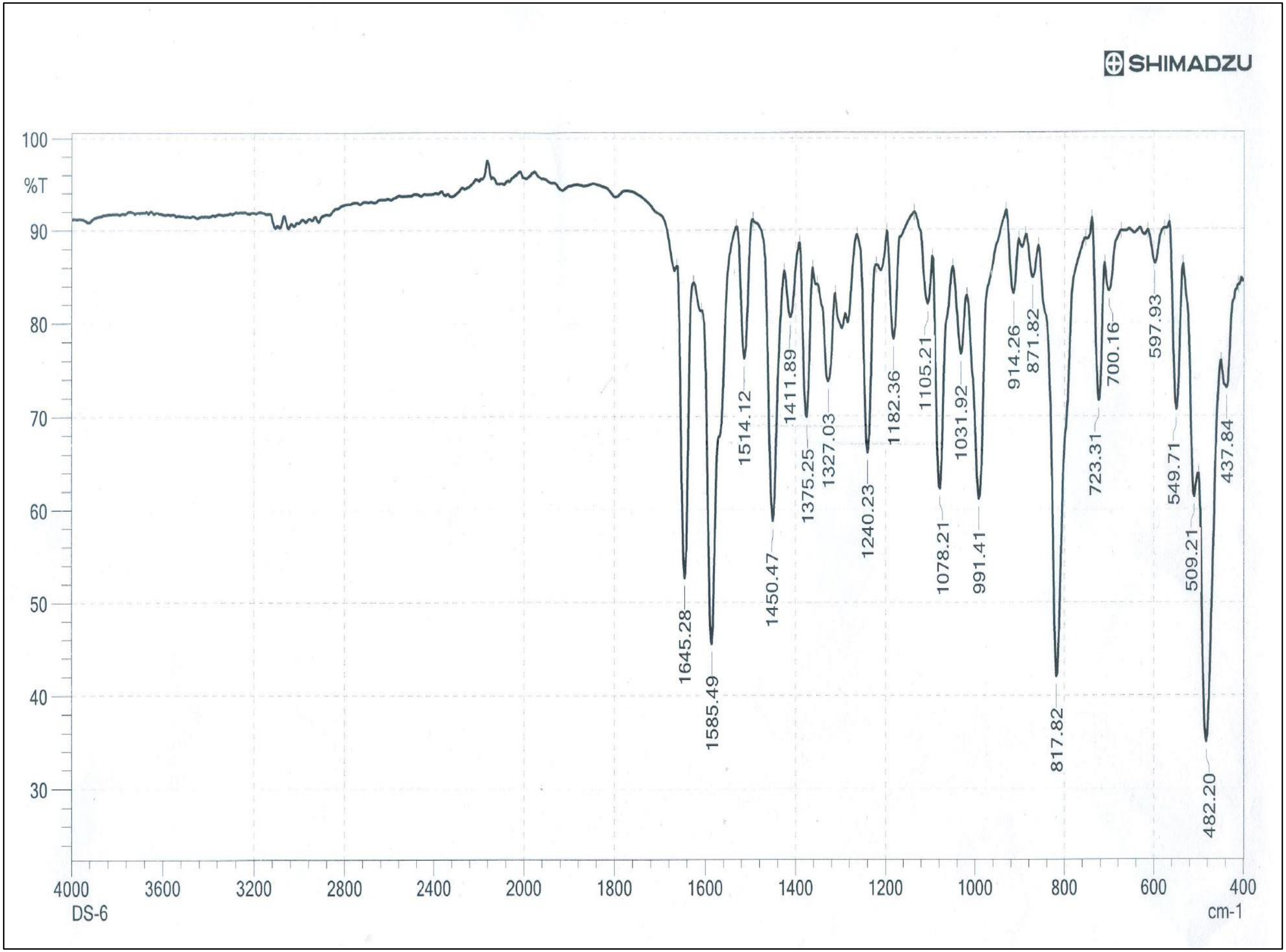

IR spectra of compound 3f

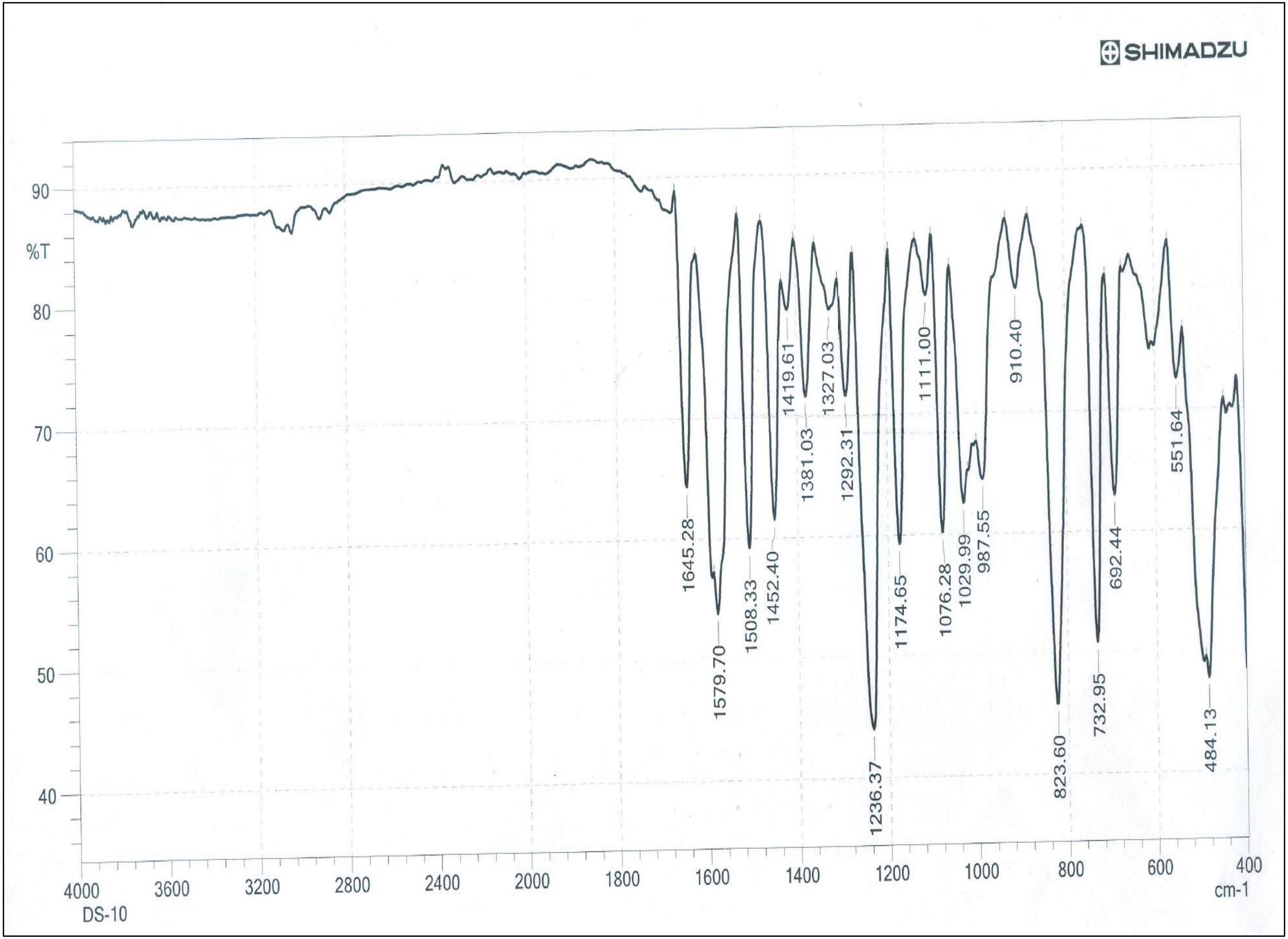

IR spectra of compound 3j

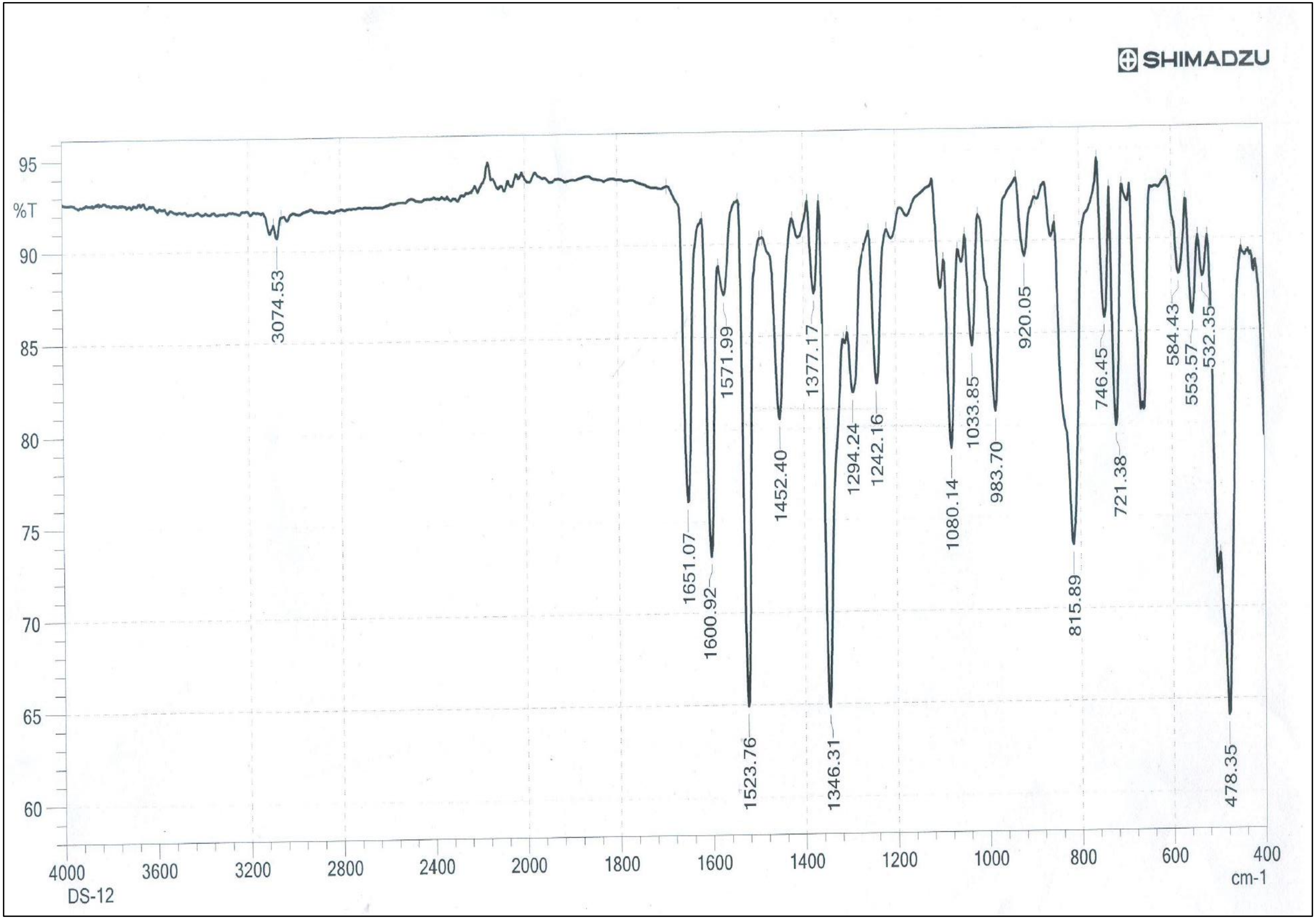

IR spectra of compound 3l

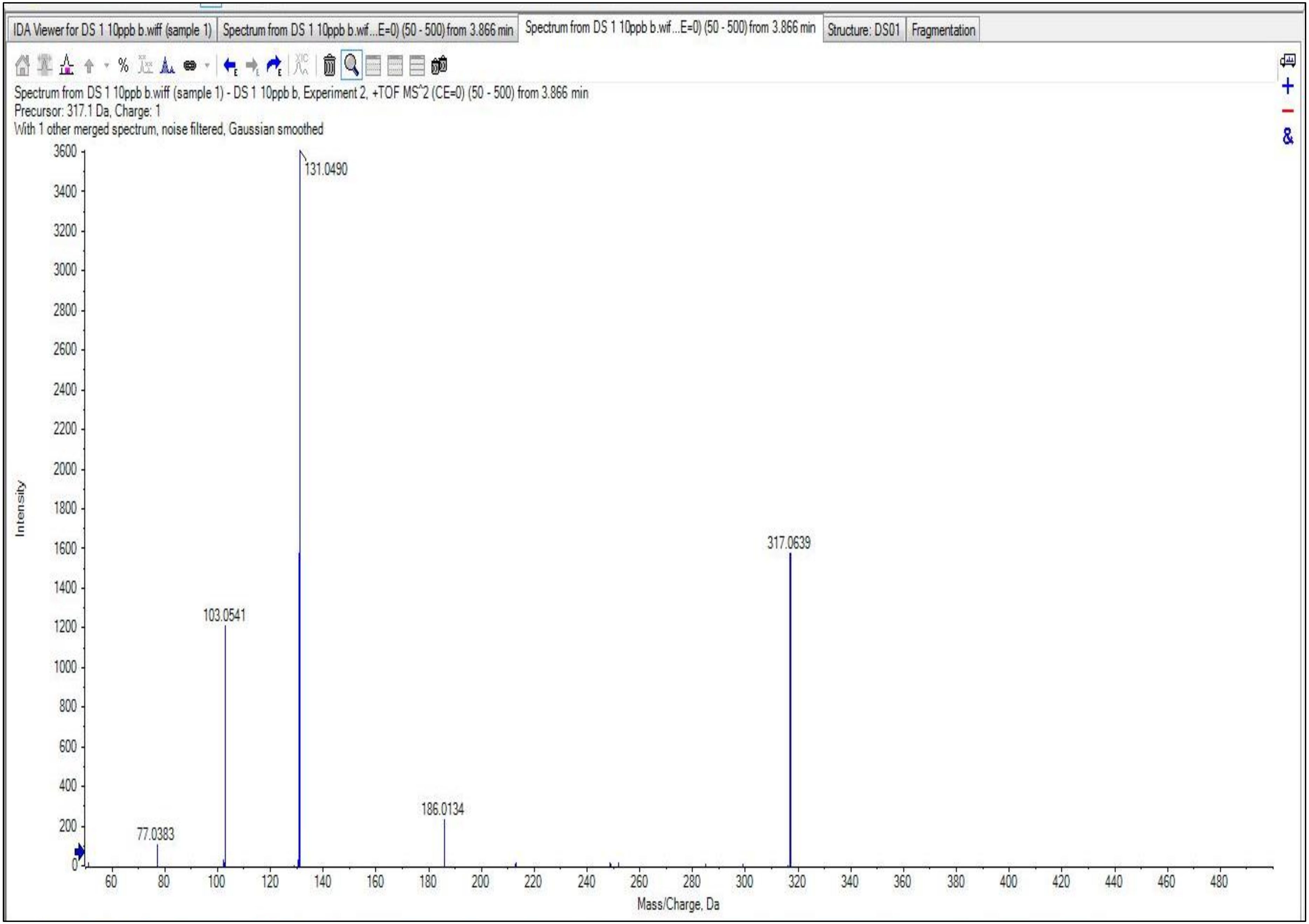

HRMS spectra of compound 3a

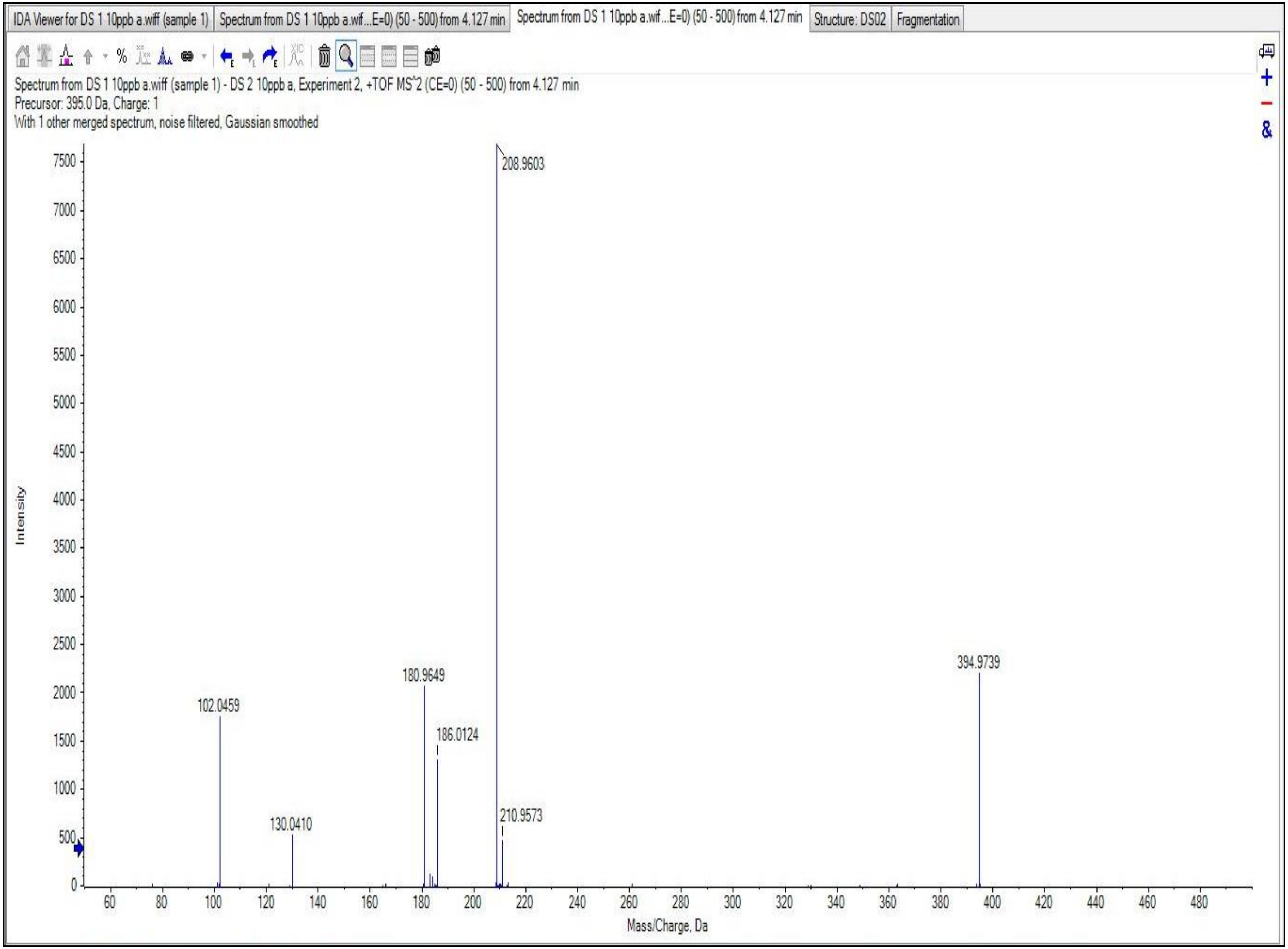

HRMS spectra of compound 3b

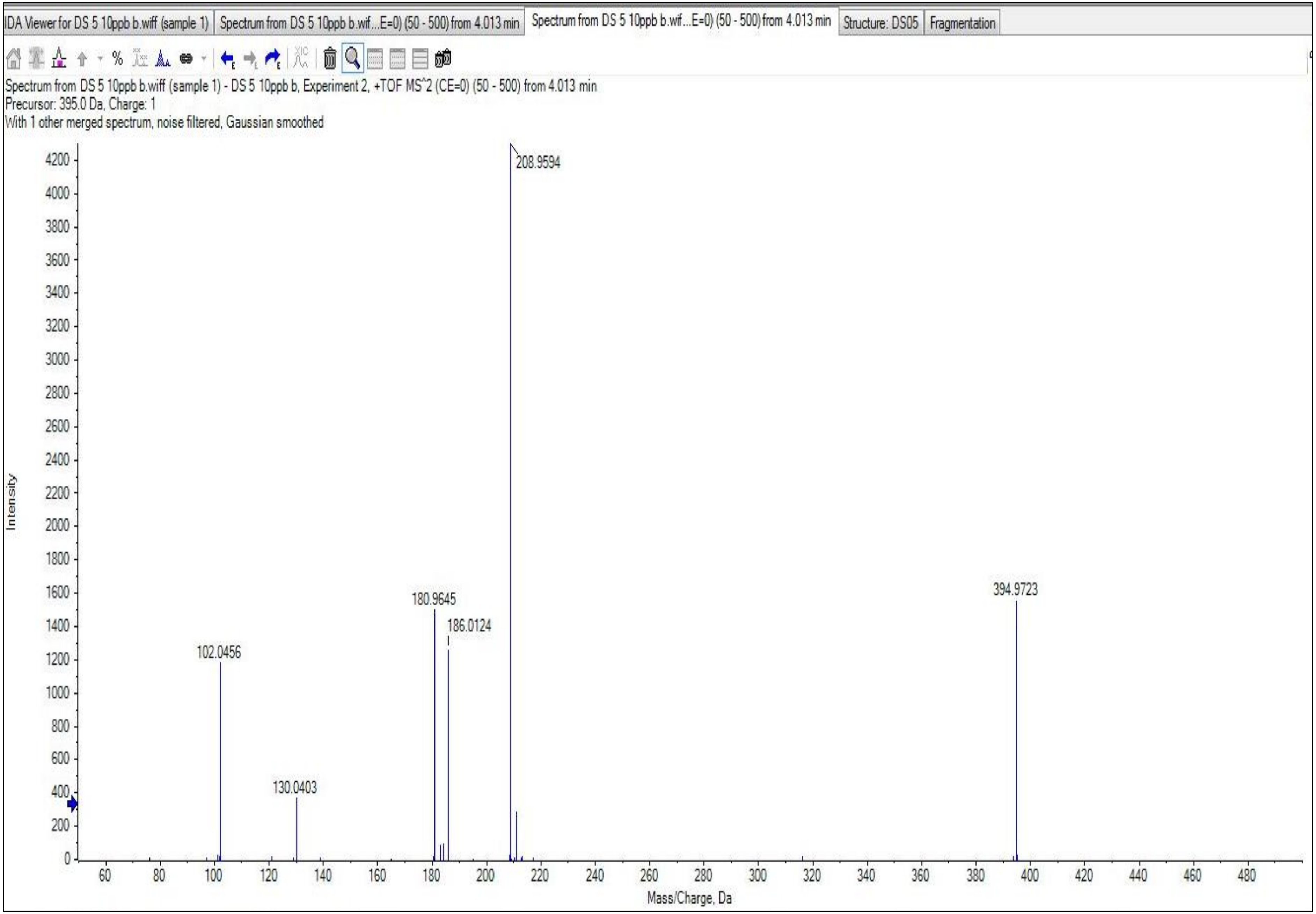

HRMS spectra of compound 3e

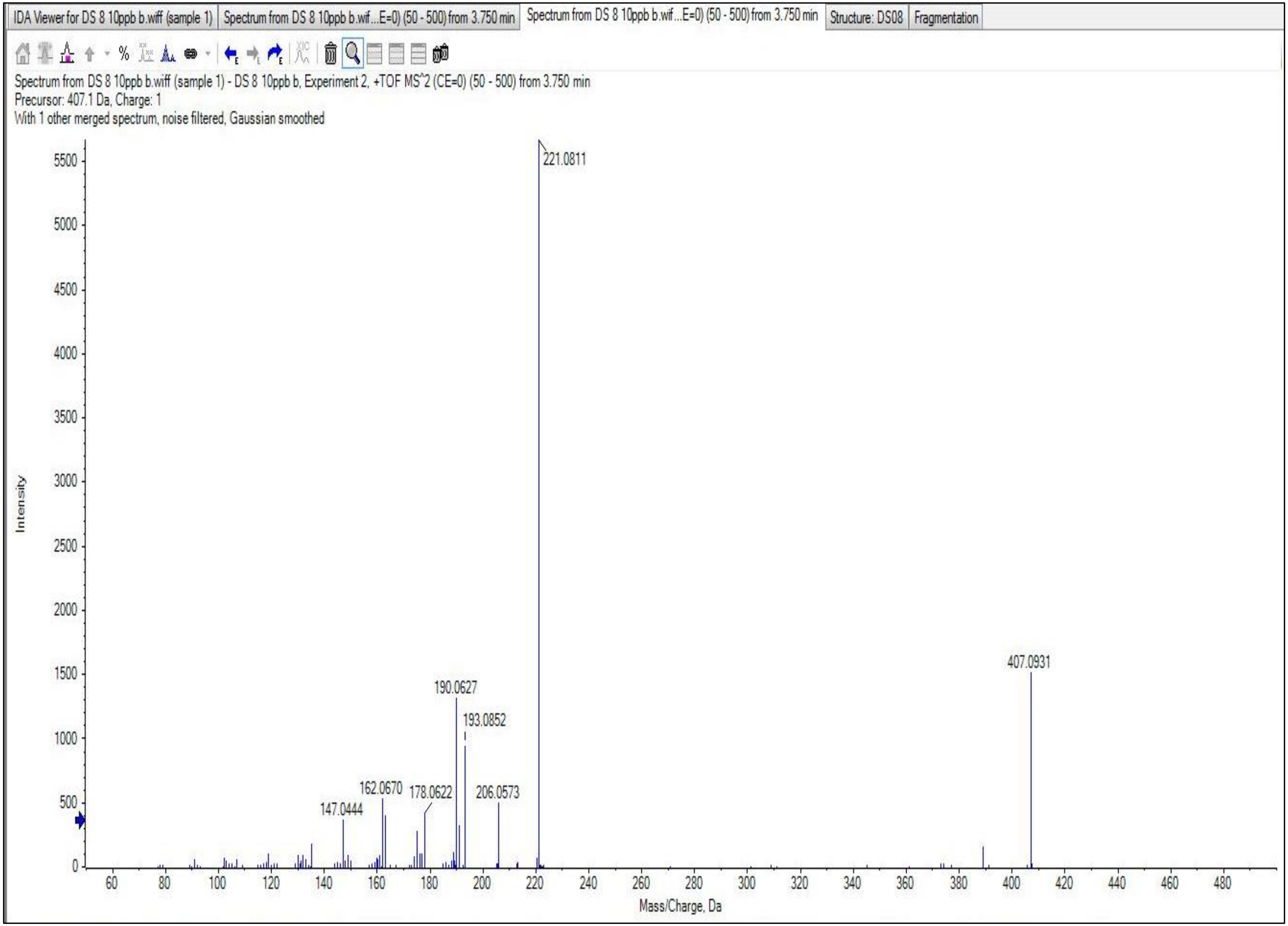

HRMS spectra of compound 3h

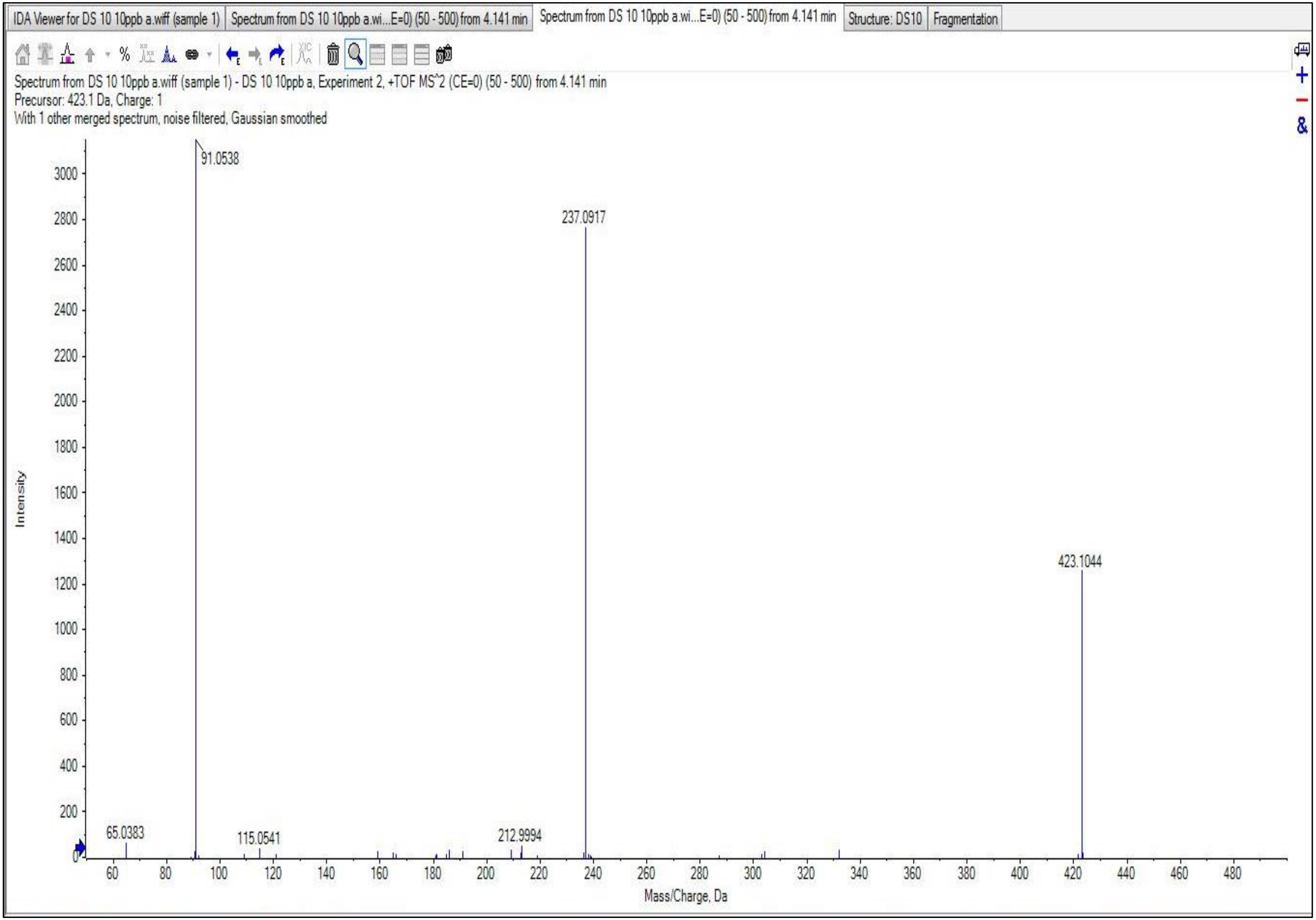

HRMS spectra of compound 3j

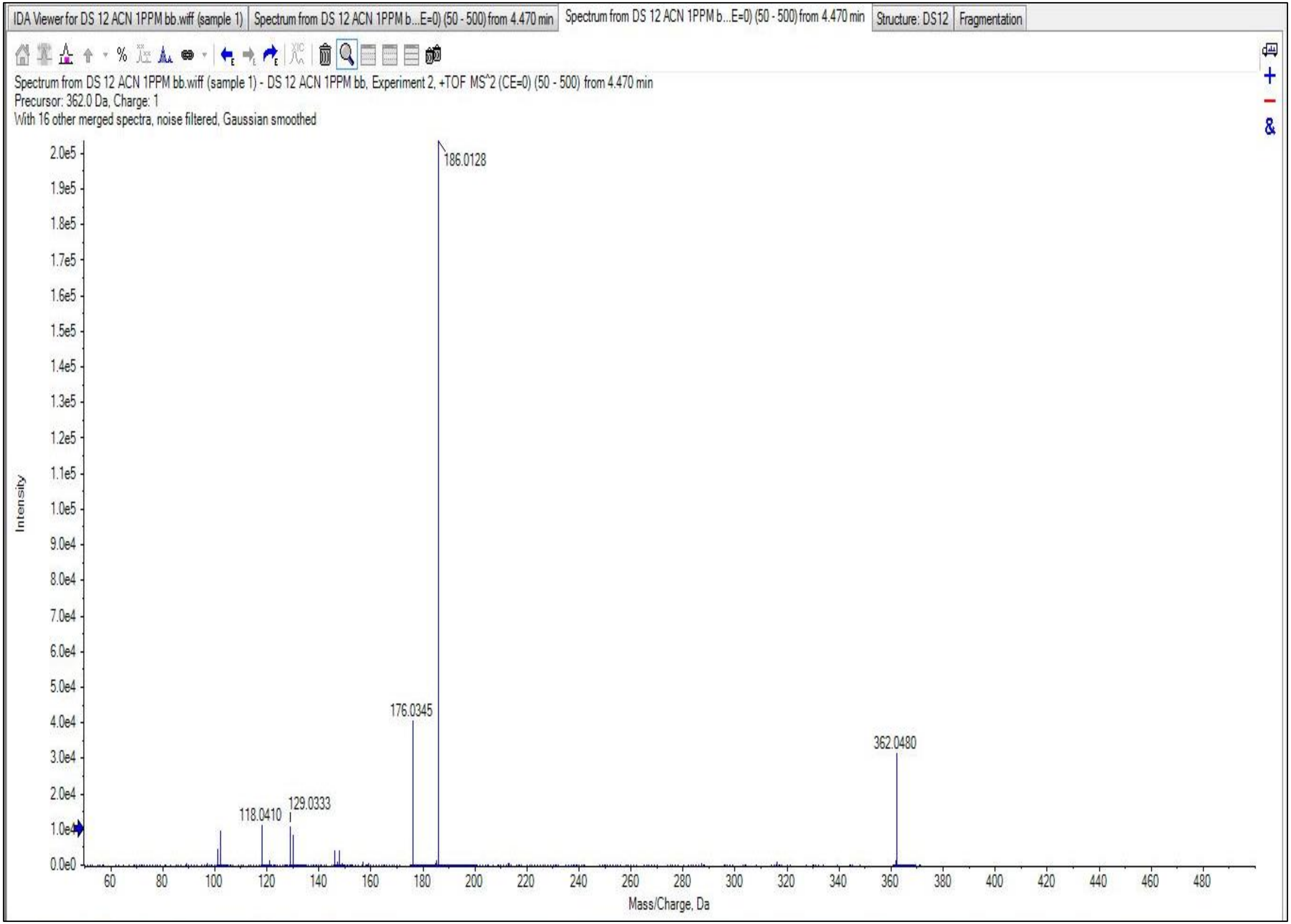

HRMS spectra of compound 3l
