# Supplementary material for: Microwave Assisted Synthesis, Characterization and Biological Activities of Ferrocenyl Chalcones and Their QSAR Analysis
Source: Front Chem. 2019 Nov 26;7:814. doi: 10.3389/fchem.2019.00814 (PMC6901998; doi:10.3389/fchem.2019.00814)
Supplement: Supplementary Data Sheet 2 — 1H-NMR, 13C-NMR, IR, and HR-MS spectra of compound synthesized by Scheme 2. [file Data_Sheet_2.PDF]

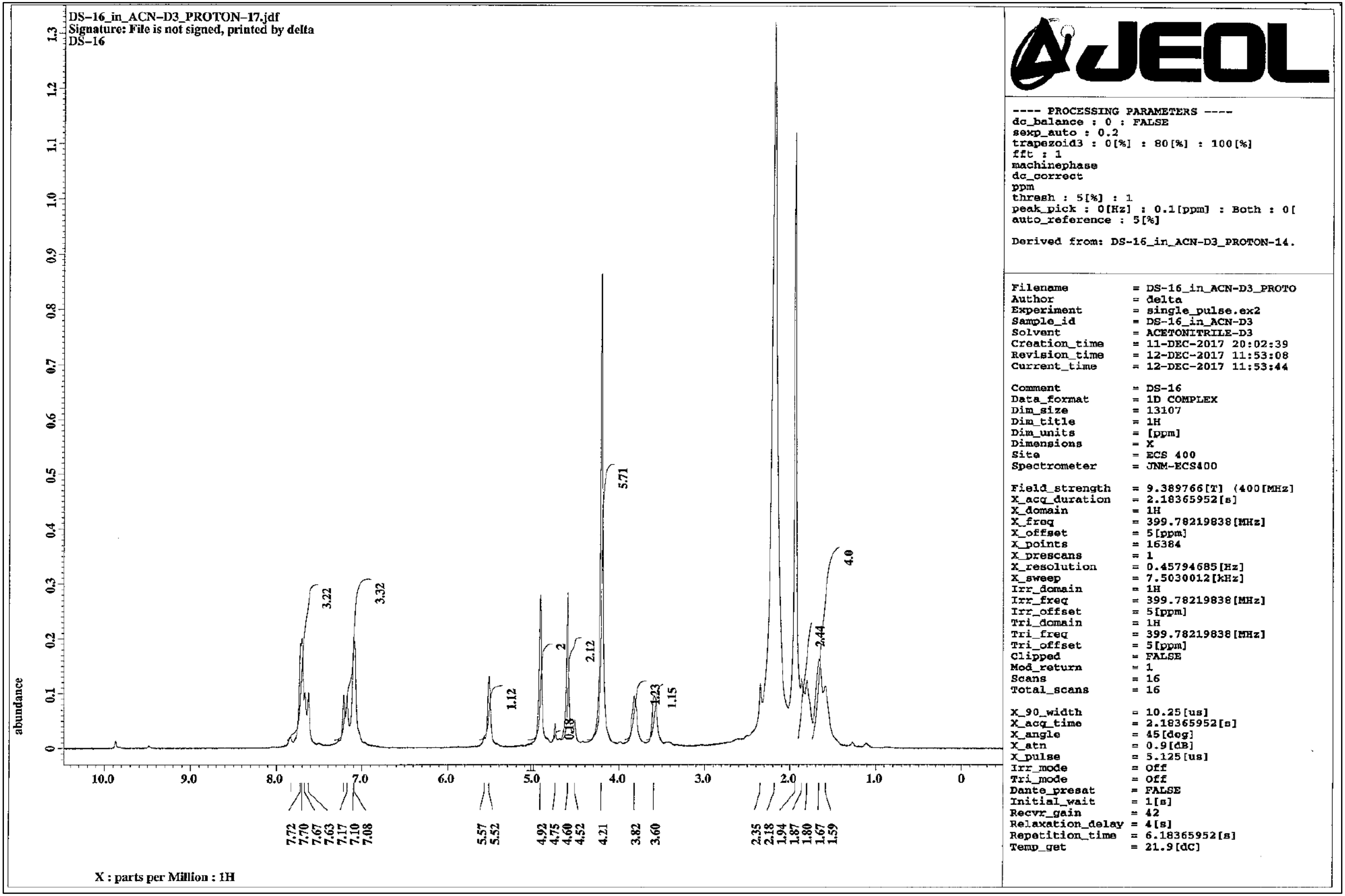


$^{1}$H NMR spectra of compound 3p

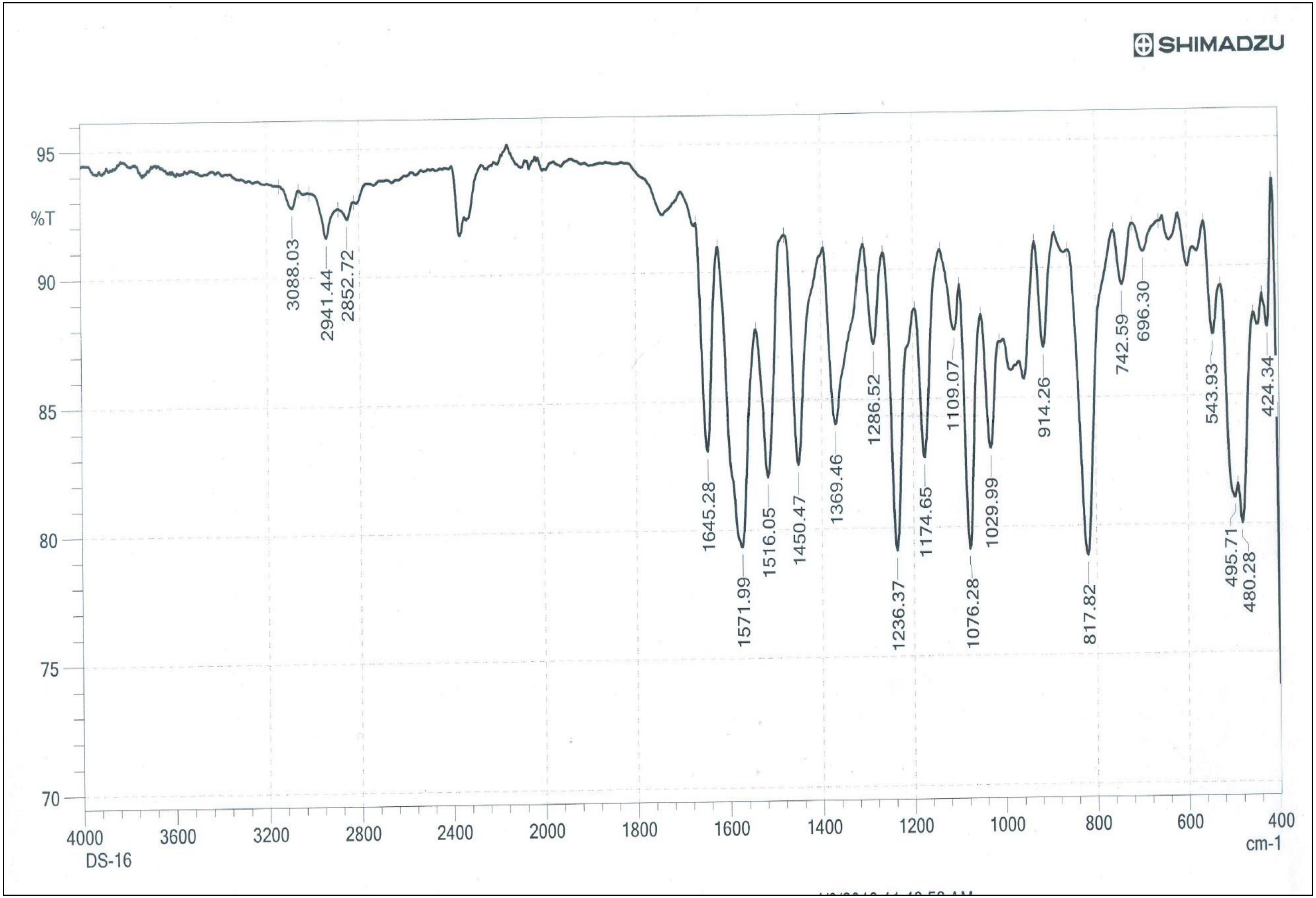


**IR spectra of compound 3p**

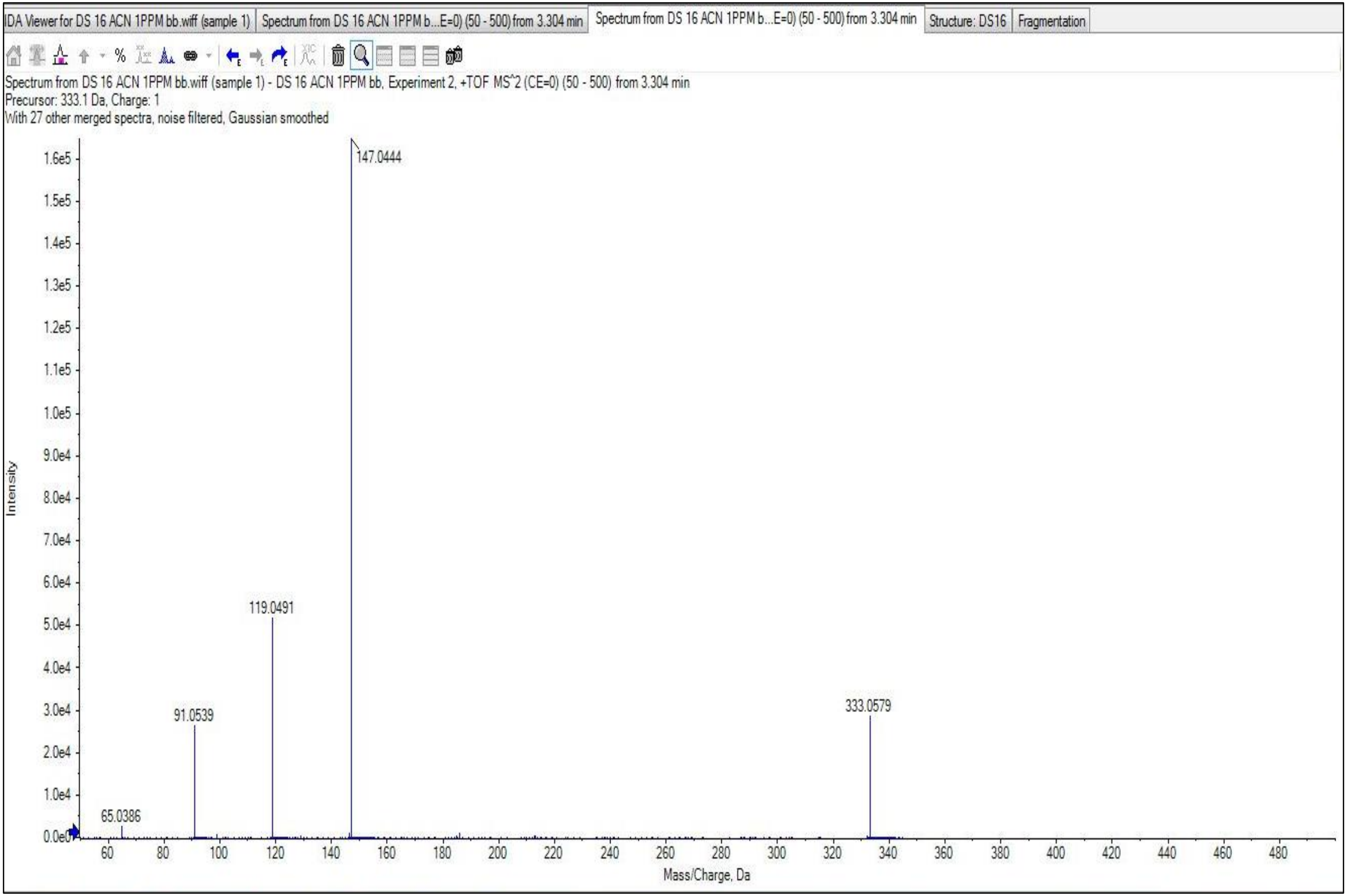


**HRMS spectra of compound 3p**
