# Supplementary material for: Microwave Assisted Synthesis, Characterization and Biological Activities of Ferrocenyl Chalcones and Their QSAR Analysis
Source: Front Chem. 2019 Nov 26;7:814. doi: 10.3389/fchem.2019.00814 (PMC6901998; doi:10.3389/fchem.2019.00814)
Supplement: Supplementary Data Sheet 3 — Enlarge view of NMR spectra. [file Data_Sheet_3.PDF]

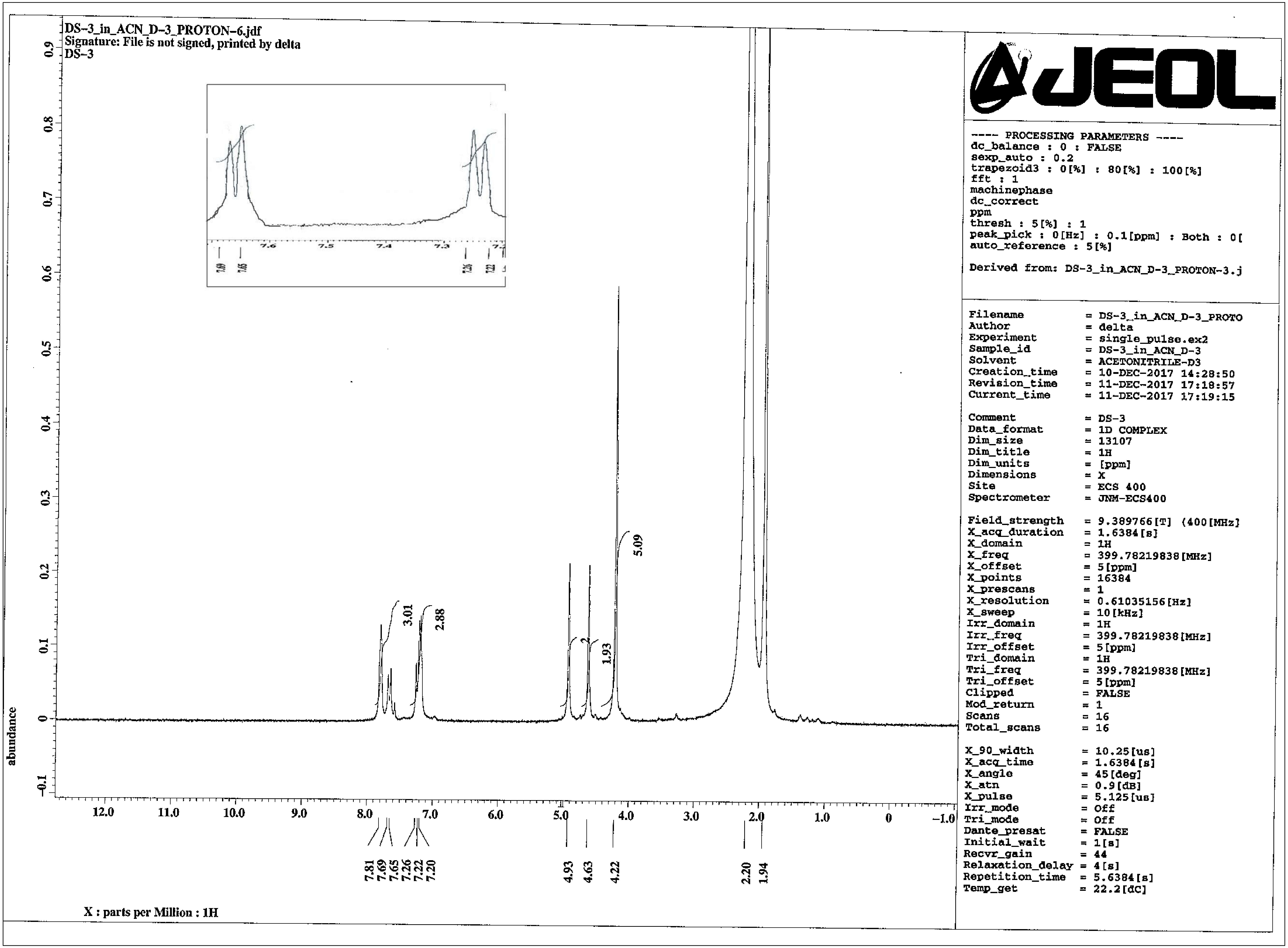

^{1}H NMR spectra of compound 3c

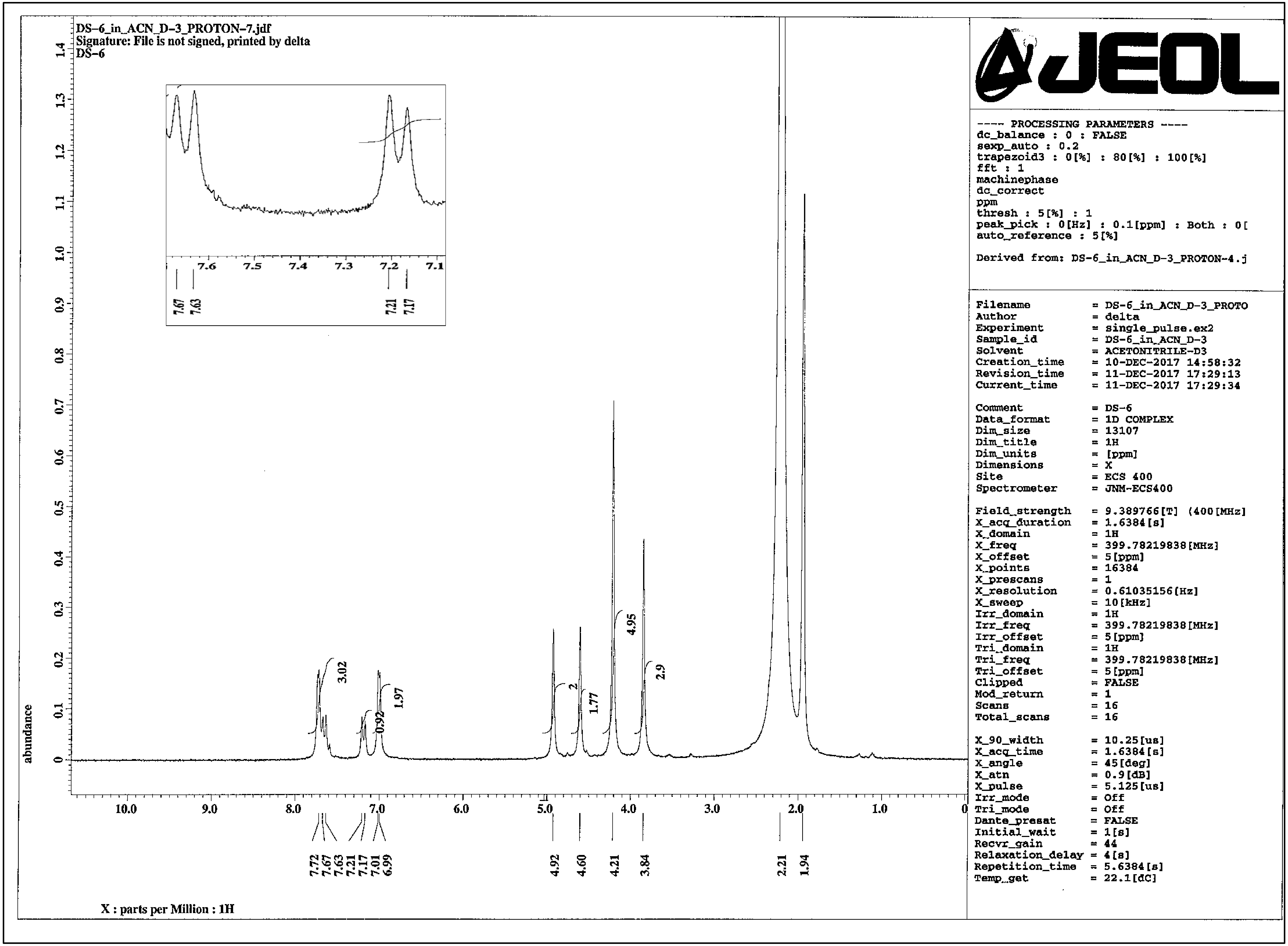

^{1}H NMR spectra of compound 3f

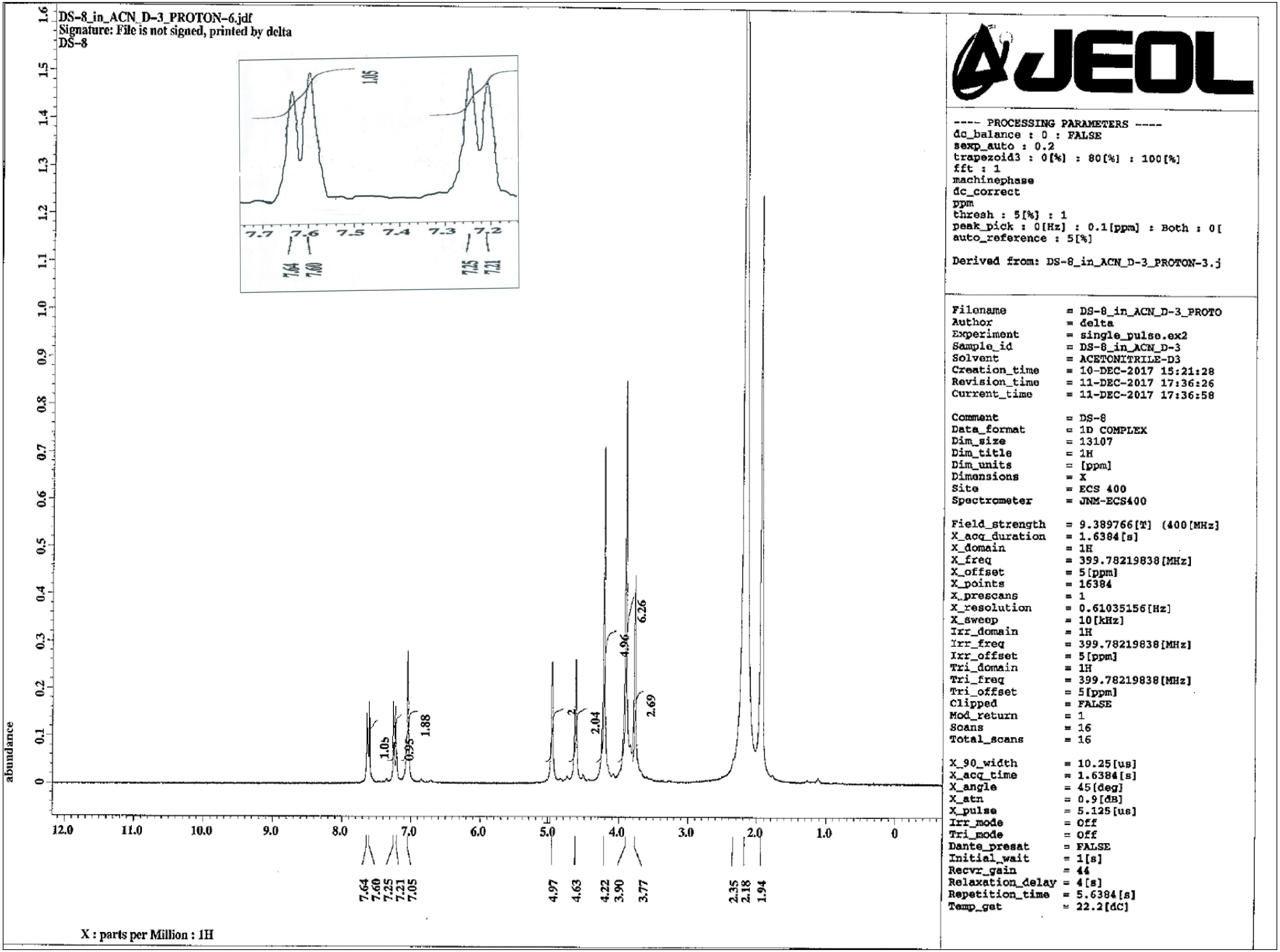

^1H NMR spectra of compound 3h

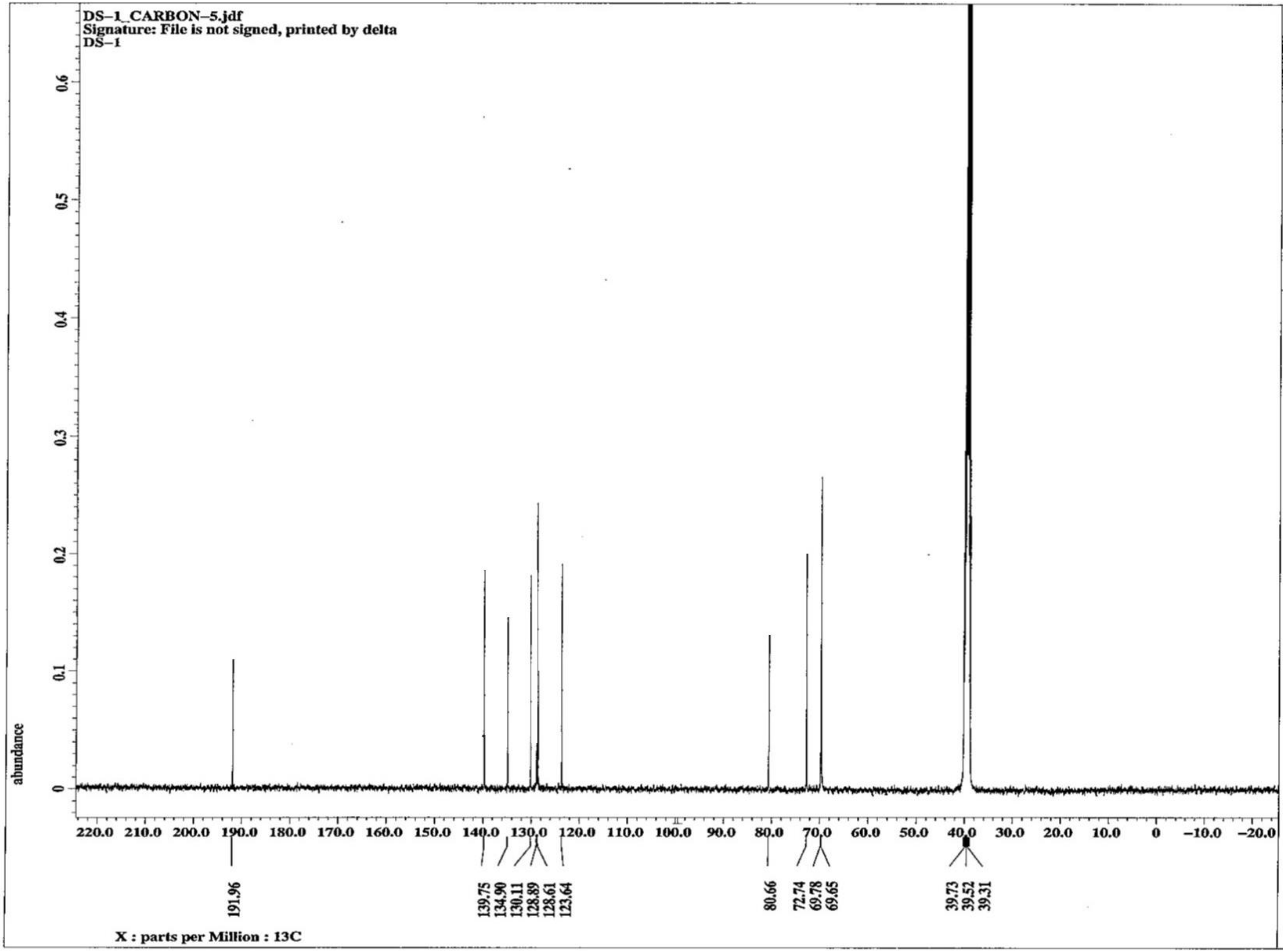

^{13}C NMR spectra of compound 3a

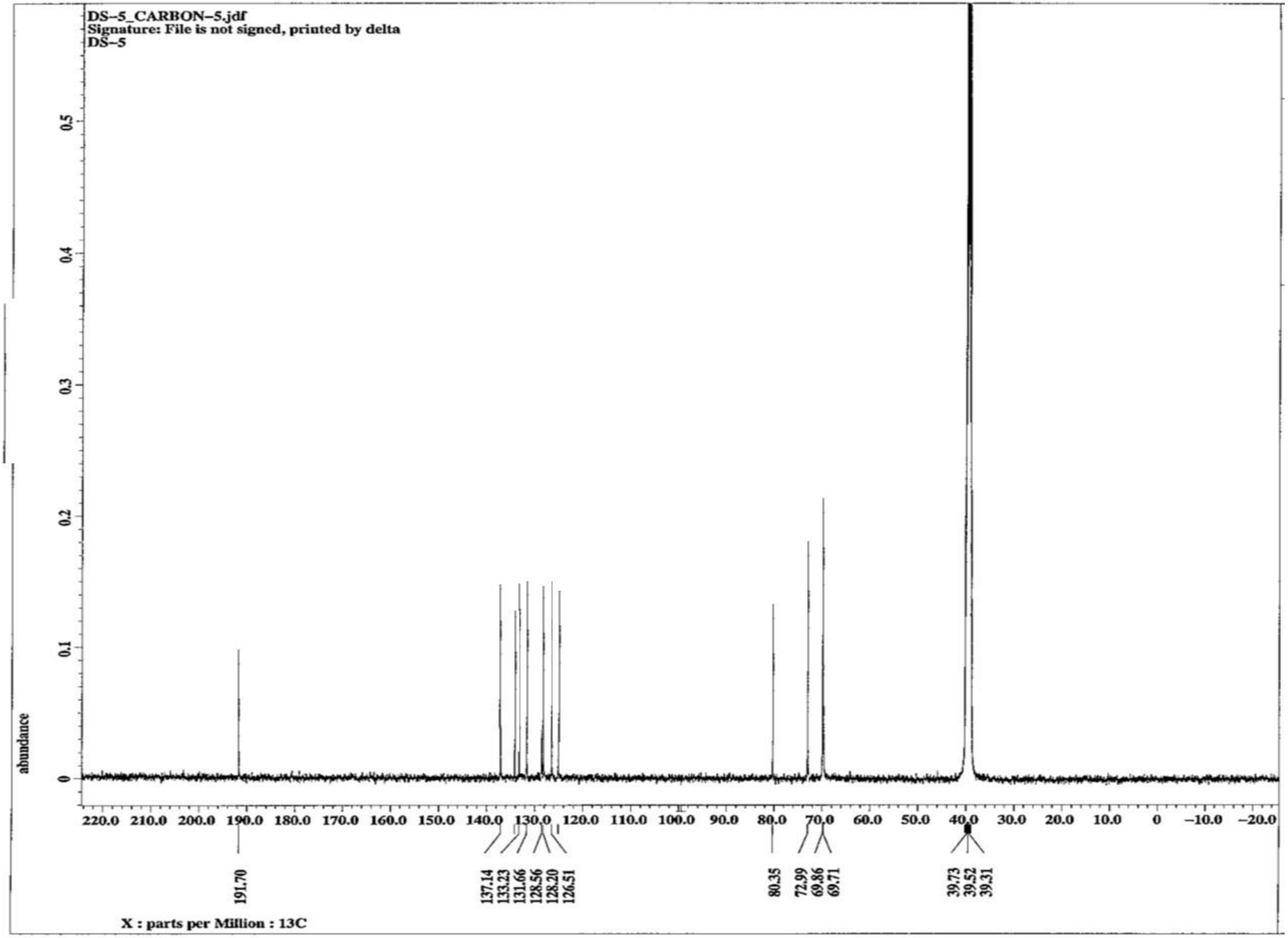

^{13}C NMR spectra of compound 3e

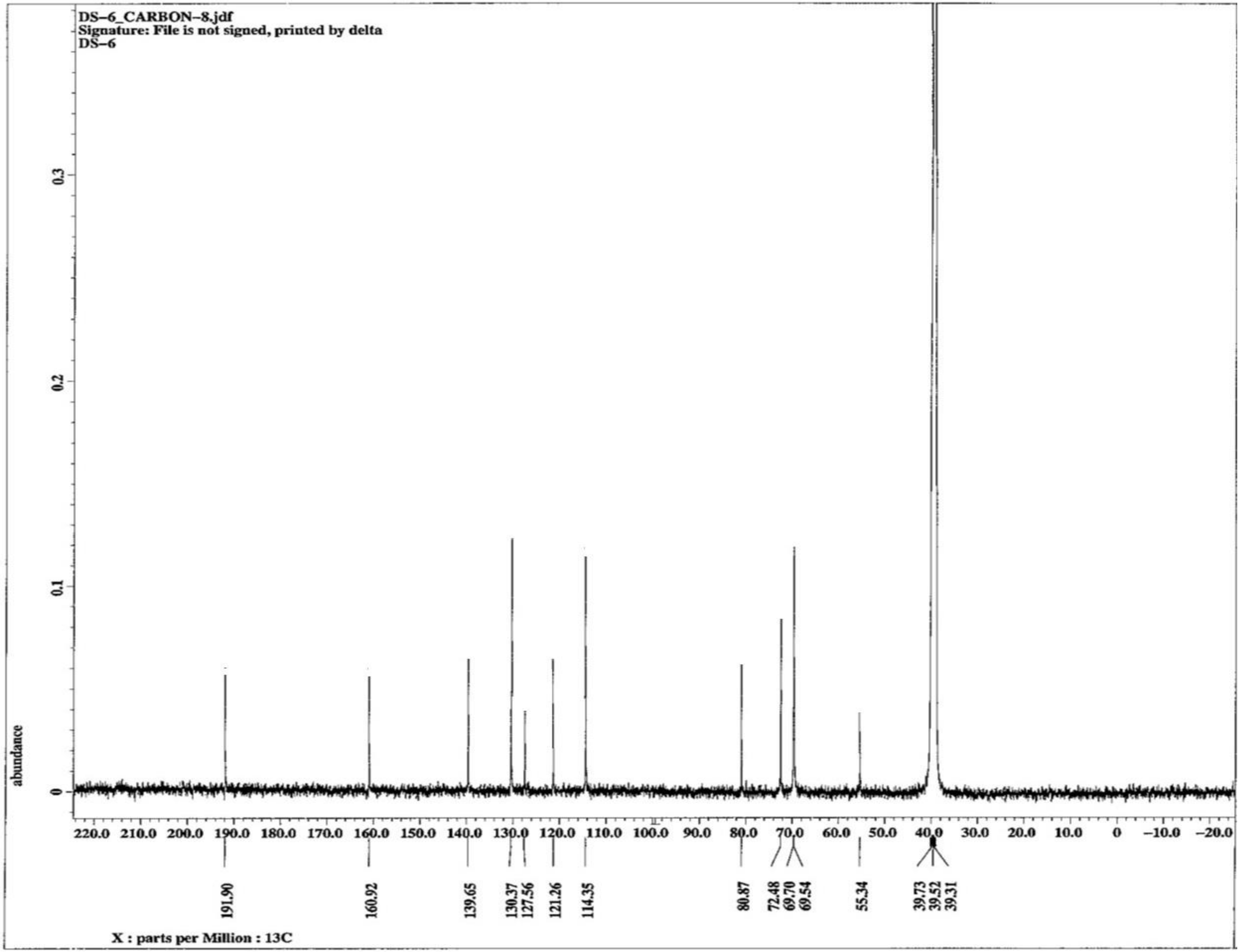

^{13}C NMR spectra of compound 3f
